# Fumonisin and Ochratoxin Production in Industrial *Aspergillus niger* Strains

**DOI:** 10.1371/journal.pone.0023496

**Published:** 2011-08-11

**Authors:** Jens C. Frisvad, Thomas O. Larsen, Ulf Thrane, Martin Meijer, Janos Varga, Robert A. Samson, Kristian F. Nielsen

**Affiliations:** 1 Center for Microbial Biotechnology, Department of Systems Biology, Technical University of Denmark, Kongens Lyngby, Denmark; 2 CBS Fungal Biodiversity Centre, Utrecht, the Netherlands; 3 Department of Microbiology, Faculty of Science and Informatics, University of Szeged, Szeged, Hungary; University of Missouri, United States of America

## Abstract

*Aspergillus niger* is perhaps the most important fungus used in biotechnology, and is also one of the most commonly encountered fungi contaminating foods and feedstuffs, and occurring in soil and indoor environments. Many of its industrial applications have been given GRAS status (generally regarded as safe). However, *A. niger* has the potential to produce two groups of potentially carcinogenic mycotoxins: fumonisins and ochratoxins. In this study all available industrial and many non-industrial strains of *A. niger* (180 strains) as well as 228 strains from 17 related black *Aspergillus* species were examined for mycotoxin production. None of the related 17 species of black Aspergilli produced fumonisins. Fumonisins (B_2_, B_4_, and B_6_) were detected in 81% of *A. niger*, and ochratoxin A in 17%, while 10% of the strains produced both mycotoxins. Among the industrial strains the same ratios were 83%, 33% and 26% respectively. Some of the most frequently used strains in industry NRRL 337, 3112 and 3122 produced both toxins and several strains used for citric acid production were among the best producers of fumonisins in pure agar culture. Most strains used for other biotechnological processes also produced fumonisins. Strains optimized through random mutagenesis usually maintained their mycotoxin production capability. Toxigenic strains were also able to produce the toxins on media suggested for citric acid production with most of the toxins found in the biomass, thereby questioning the use of the remaining biomass as animal feed. In conclusion it is recommended to use strains of *A. niger* with inactive or inactivated gene clusters for fumonisins and ochratoxins, or to choose isolates for biotechnological uses in related non-toxigenic species such as *A. tubingensis, A. brasiliensis, A vadensis* or *A. acidus*, which neither produce fumonisins nor ochratoxins.

## Introduction

*Aspergillus niger* is one of the most important industrial filamentous fungal species used in biotechnology [1]–[2] where it is used extensively for organic acid production [3]–[5] and for the production of extracellular enzymes [6]. Other applications include: biotransformation of xenobiotics [7], bioremediation and waste pretreatment [8]–[9], and cell protein for feed [10]–[11]. Further significant applications include its use as transformation host of heterologous proteins and secondary metabolites due to its high growth rate, low-pH tolerance [5], [12]–[13], and high polyketide production rate [14].

Three strains of *A. niger* have already been full genome sequenced: ATCC 1015, NRRL 3 and CBS 513.88 [1]–[2], [15]. These strains have subsequently been examined intensively using transcriptomics and metabolomics to explore and understand growth, differentiation, chemistry and physiology of the species [1], [14], [16]–[20]. All three strains have a fumonisin gene cluster similar to the one responsible for the production of the known fumonisin mycotoxins by *Fusarium verticillioides* ( =  *Gibberella moniliformis*) [21]–[22]. *A. niger* strains produce fumonisins B_2_, B_4_, and B_6_ but not FB_1_ [23]–[24]. Surveys of isolates from coffee, grapes and raisins have shown that many produce fumonisins strongly indicating that *A. niger* is responsible for the presence of low amounts of FB_2_ in almost a quarter of all examined wine samples [25]–[26] as well as in half the examined raisins and grapes [27]–[29].

No surveys on fumonisin and ochratoxin A (OTA) production capability have been made in industrial strains. Depending on the commodity or environment surveyed [30]–[32], Since up to 41% of all *A. niger* isolates produced ochratoxins and 75% produced fumonisins [30], [33], there is a risk that industrial strains could also produce those mycotoxins.

A number of black Aspergilli have been misidentified, among other because of changing taxonomies. It is therefore important to examine strains in all black *Aspergillus* species for mycotoxin production. Furthermore they should be identified using a polyphasic taxonomic approach which is now the primary biosystematic approach for *Aspergillus*. This approach includes a combination of morphological, physiological, chemical and molecular methods, decreasing the risk of misidentification of strains [34]–[36]. Even though *A. niger* has been given GRAS (generally regarded as safe) status in numerous industrial processes, the potential for mycotoxin production by industrial strains of this species is a serious problem as the ability to produce the mycotoxins was not known at the time when the *A. niger* processes were granted GRAS status [37]. It needs to be mentioned that numerous batches of all products have been tested for cytotoxicity, carcinogenicity and other tests for product approval. These are all tests which clearly would have picked up significant concentrations of ochratoxins and/or fumonisins, indicating that these toxins may not be produced under industrial submerged growth conditions [37]. However media, growth conditions and strains are constantly being changed and improved, indicating a continuous risk of presence of OTA and fumonisins in the final products.

Because there are no large surveys on mycotoxin production in industrial black Aspergilli available and increasing evidence is emerging on its production of fumonisins and OTA on food products, we decided to study production capability of a large number of strains. Focus was on industrial strains, but for completeness strains from the remaining 17 related black *Aspergillus* species were included. Some fumonisin and ochratoxin A producing *A. niger* strains were also tested for mycotoxin production when grown in media suggested for citric acid production.

## Materials and Methods

Water was purified from a Milli-Q system (Millipore, Bedford, MA). Certified standards solutions consisting of 50 µg/mL of fumonisin B_1_ (FB_1_) and 50 µg/mL fumonisin B_2_ (FB_2_) were obtained from Biopure, Tulln, Austria. Fumonisin B_3_ (FB_3_) was a gift from Dr. Michael Sulyok, Center for Analytical Chemistry (IFA-Tulln, Austria) and other reference standards including FB_4_ and FB_6_ were available from previous studies [24].

### Fungal materials

Strains were inoculated in three points on CYA agar with 5% NaCl (CYAS) [38] and YES agar [36]. Biokar yeast extract was used in CYA, YES and CYAS. The fungi were incubated at 25°C for 7 days in darkness before extraction. The identity of all strains examined was confirmed by polyphasic identification [34] including partial sequencing of beta-tubulin and calmodulin genes.

To test production on media suggested for citric acid production, five *A. niger* cultures (NRRL 567; IBT 19558; ITEM 7097; CBS 101705 were inoculated (using 2 agar plugs from a one week old culture on CYA agar grown at 25°C) into 300 ml Erlenmeyer flasks with baffles containing 100 ml medium of the media CIT1 [39] pH 2.5, CIT2 [40] pH 3.0, CIT3 [41] pH 3.0, CIT 4 [42] pH 4.1, and CIT 5 [43] pH 5.0. Flasks with CIT 1, 2 & 3 were incubated in a rotary shaker (150 rpm) at 30°C in darkness for one week, while CIT 4 & 5, both containing 30 ml methanol pr liter medium, were still cultures incubated for one week at 25°C in darkness. Secondly, 9 strains of *A. niger* (CBS 126.48, 101705, IBT 19558, NRRL 3, 330, 350, 567, 599, 3122), were inoculated in still culture (100 ml medium in 300 ml flasks) using a spore suspension of 10^4^ conidia/ml and were cultured at 25°C for 8 days. The same 9 strains were inoculated into the same media (10^41^ conidia/ ml in100 ml in baffled 300 ml flasks), but incubated at 30°C for 8 days and shaken at 75 rpm. Control flasks with medium only were also incubated for 8 days at 25°C. All experiments were done in duplicate.

### Fumonisin analysis

Extraction of fumonisins was accomplished by by adding 750 µl of 75% methanol in water to five agar plugs followed by ultrasonication (Branson 1510) for 50 min. The extraction liquid was filtered through a 0.45 µm polytetrafluoroethene (PTFE) filter.

Liquid media (CIT 1–5) were centrifuged at 12,000 g and the supernatant analyzed directly. The biomass was washed twice with 10 ml water, ultrasonicated for 3 hr in 10 ml 75% methanol, centrifuged for 5 min at 12,000 g and the supernatant analyzed.

**Accurate mass LC-MS** was performed on an Agilent 1100 system equipped with a photo diode array detector (DAD) and coupled to a LCT orthogonal time-of-flight mass spectrometer (Waters-Micromass, Manchester, UK) operated in positive ESI^+^ mode [14]. Separation was done on a 50×2 mm i.d., 3 µm, Luna C_18_ II column (Phenomenex, Torrance, CA) column using a water-acetonitrile gradient system starting with 0.3 ml/min flow of 30% acetonitrile, which was increased linearly to 60% in 5 min, then to 100% while also increasing the flow to 0.5 ml/min, holding this for 2 min [14]. A FB_1_ and FB_2_ mixture was analyzed every ca. 50 samples along with blank sample. Reference standards of FB_3_, malformins A_1_, A_2_ and C, and asperazine were also co-analyzed in the sequences.

Fumonisins were detected from the reconstructed ion chromatograms of the [M+H]^+^ ion ±*m/z* 0.01. Other metabolites were detected as the predominant ion in extracted ion chromatograms (±*m/z* 0.01). All peaks were matched against an internal database of 850 standards and Antibase 2009 (John Wiley and Sons inc.).

### LC-MS/MS analysis

Selected samples where FB_2_ was not detected or found in trace amounts, were analyzed by LC-MS/MS on an Agilent 1100 liquid LC system coupled to a Quattro Ultima triple mass spectrometer (Micromass). The separations were performed on a Gemini C_6_-phenyl column (Phenomenex, 50×2 mm, 3 µm). Separation was performed using a linear gradient starting from 20% acetonitrile in water (both 20 mM formic acid) to 55% acetonitrile for 6 minutes at a flow rate of 0.3 ml/min, which was increased to 100% acetonitrile in 30 sec and a flow of 0.5 ml/min keeping this for 3.5 min. Tandem mass spectrometry was performed in ESI^+^ MRM [14], [24] searching for FB_1–4,6_ as well as malformins and OTA.

### Citric acid analysis

The centrifuged broth of CIT 1–5 were analyzed for citric acid using reversed phase LC on an Agilent 1100 system equipped with a DAD and a 150×2 mm i.d., 4 µm, Synergi MAX-RP C_18_ column (Phenomenex). Citric acid was eluted at 25°C using a flow of 0.3 ml/min water buffered with 500 µl/l trifluoroacetic acid. After 10 min acetonitrile was increased linear to 100% in 3 min, keeping this 3 min at a flow rate of 0.5 ml/min, reverting to 100% water in 3 min, equilibrating for 5 min. Sub-samples of 0.3 µl were injected, and samples quantified by external standard calibration.

## Results

### Black *Aspergillus* species producing fumonisin B_2_ and ochratoxin A

Available strains of the black Aspergilli which have been used in the bioindustry, freshly isolated strains, and strains obtained from culture collections were confirmed for identity or re-identified using a polyphasic approach [34]–[36] (beta-tubulin and calmodulin sequencing, HPLC-UV-MS profiling, microscopy, physiology). Most biotechnological isolates were indeed *Aspergillus niger sensu stricto*, however some strains (23%) originally identified as *A. niger* were re-identified as *A. tubingensis*, *A. brasiliensis*, *A. acidus* or *A. carbonarius* (Table S1). Identification of a large number of strains from soil, foods and other habitats showed that *A. niger* was most common among the black Aspergilli (44%), but that *A. tubingensis* was approximately half as common (20%), while *A. acidus* (10%), *A. carbonarius* (7%) *A. brasiliensis* (4%) were less common, and the remaining species in the black Aspergilli were rare (below 1%). Among the industrial strains used (Table S2, S3, S4) most isolates (70%) belonged to *A. niger*, while *A. tubingensis* accounted for 12%, *A. acidus*, *A. carbonarius* and *A. brasiliensis* for each 6%, and *A. vadensis* for 2%. Recent results indicate that *A. niger* may be subdivided into two clades represented by *A. niger sensu stricto* and *A. awamori* [28]. If the *A. awamori* clade is accepted as a valid species, some of the best producers of fumonisin are *A. awamori*, including the citric acid producer NRRL 567 (Table S1, Table S2). In the current study of black Aspergilli we have not found fumonisin B_1_ or B_3_ in any strain yet.

Apart from *A. niger*, none of the other 17 species in *Aspergillus* section *Nigri* produced detectable amounts of fumonisins on CYAS agar (Table 1) with one exception: the culture ex type of *A. lacticoffeatus* produced fumonisin B_2_ and OTA. However, beta-tubulin and calmodulin sequencing of *A. lacticoffeatus* indicated it is a colour mutant of *A. niger* (data not shown). The frequency of fumonisin producers in *A. niger* was 81% (180 strains tested) and the strains producing it included well known NRRL strains suggested for use in citric acid production [42] and for many other biotechnological purposes (Table S2, Table S3). On the CYAS medium 158 strains of *A. niger* were tested positive for production of FB_2_, with most also producing FB_4_ and FB_6_, although FB_2_ was always the major fumonisin compound. In average the ratio of FB_2_∶FB_4_ was 8, and the ratio FB_2_∶FB_6_ was 200. The frequency of *A. niger* producing OTA was 17% on YES agar, and only 10% produced both toxins (results for each strain in Table S1), indicating no correlation between the production of the two toxins.

**Table 1:** Detection of fumonisin B_2_ and ochratoxin A by the 18 species in *Aspergillus* section *Nigri* (species in bold are used in biotechnology).

Species	No. of strains producing detectable fumonisin B_2_	No. of strains not producing detectable fumonisin B_2_	No. of strains producing detectable ochratoxin A	No. of strains not producing detectable ochratoxin A	No. of strains producing both fumonisin B_2_ and ochratoxin A
***A. acidus***	0	42	0	42	0
*A. aculeatinus*	0	14	0	14	0
***A. aculeatus***	0	13	0	13	0
***A. brasiliensis***	0	18	0	18	0
***A. carbonarius***	0	30	30	0	0
*A. costaricaensis*	0	1	0	1	0
*A. ellipticus*	0	2	0	2	0
*A. heteromorphus*	0	1	0	1	0
*A. homomorphus*	0	2	0	2	0
*A. ibericus*	0	3	0	3	0
***A. japonicus***	0	4	0	4	0
***A. niger***	145	35	30	150	18
*A. piperis*	0	1	0	1	0
*A. sclerotiicarbonarius*	0	6	0	6	0
*A. sclerotioniger*	0	1	1	0	0
***A. tubingensis***	0	83	0	83	0
*A. uvarum*	0	5	0	5	0
***A. vadensis***	0	1	0	1	0
**Industrial ** ***A. niger***	28	9 (4 OTA)	12	25	8

Among the industrial strains (n  =  69) of *A. niger*, 83% produced fumonisin B_2_, 33% produced OTA and 26% produced both toxins (Table 1; Table S1), again showing no correlation between the production of the two toxins. However OTA production was more common among the industrial strains. Three of the most popular industrial strains (NRRL 337, 3112, and 3122) produced both mycotoxins. All the strains of black Aspergilli, that are used often, NRRL 3 (92 papers), NRRL 2270 (75 papers), NRRL 599 (31 papers) are *A. niger* and they produce fumonisins (Table S2). The NRRL 3 Δ*pyrG* mutant (N402) still produced fumonisins, and likewise all mutants or otherwise genetically modified strains maintain the fumonisin production capability. The only exception was CBS 114.50, a yellow mutant derived from CBS 113.50 (Table S3).

Only five strains of *A. niger*, used or suggested for use in biotechnology did not produce either fumonisins or OTA (Table S2), and none of these are commonly used. Four other black *Aspergillus* species have been commonly used for biotechnology under the name *A. niger*: *A. acidus, A. brasiliensis, A. tubingensis*, and *A. vadensis* (Table S2). Thus 25% of the industrial strains called *A. niger* were misidentified according to the most recent taxonomy [34]. Only few strains of *A. carbonarius* have been used by biotechnological industries and these all produced OTA [34], [36], [44].

Alternative non-mycotoxigenic species that may be used in biotechnology instead of *A. niger* includes: *A. acidus*, *A. brasiliensis*, *A. tubingensis*, and *A. vadensis*, while non-toxinogenic species that are closely related to *A. carbonarius* include *A. ibericus* and *A. sclerotiicarbonarius* [34].

### Mycotoxin production by *A. niger* on media suggested for for citric acid production

Nine *A. niger* strains which produced fumonisins and OTA on CYAS agar were tested on five media (CIT1-5) reported to be optimal for citric acid production [39]–[43].

Based on these we decided to examine mycotoxin production in depth in three of these media using 9 strains of *A. niger* (of which 7 are used in biotechnology).

All strains produced citric acid in CIT4, both under still and shake conditions (12–34 g/l), and under these conditions all strains produced fumonisins, except 3 which produced no or trace amounts of FB_2_ (Table S5). NRRL 567 produced the highest amounts of FB_2_ (0.16–3.3 mg/l in the broth and 0.23–3.4 mg/kg in the mycelium), while CBS 126.48 produced the highest amounts of OTA (∼0.1 mg/l in broth, ∼2 mg/kg in the mycelium). In all cases more toxin was detected in the mycelium than in the broth, and toxins were produced both under still and shake conditions. Malformins C, A_1_, and A_2_ were produced in liquid culture by all isolates which could produce these on agar media. Fumonisins and OTA were also produced in the media CIT2 (Table S6) and CIT5 (Table S7). There appeared to be no correlations between high amounts of sporulation, citric acid and high amounts of fumonisins or OTA.

## Discussion

The proportion (83%) of fumonisin producing strains among the industrial strains was a little higher than that found on strains isolated from coffee (76% [33]) or from raisins (77% [30]) and among the non-industrial strains, 81% were producers. These similar fumonisin frequencies suggest that the fumonisin producing capability is not correlated to general growth rate or metabolism, secretion or other important biotechnological features as a result of industrial domestication of *A. niger*. All evidence reported here indicates that *A. niger* strains are capable of producing fumonisin B_2_, B_4_ and B_6_ (Table S3). A recent report that *A. niger* can also produce fumonisin B_1_ and B_3_ [28] has been questioned by other authors, based on both genetic and analytical evidence [27], [29].

However, 33% of the industrial strains produced OTA, as opposed to non-industrial strains of which 7% produced OTA. This higher percentage in the industrial strains could have two reasons: i) production fitness is generally higher in OTA producing strains so these have been selected more frequently or; ii) there are differences in the distribution of ochratoxin producing isolates between various habitats, since frequencies between 0 and 41% have been reported, with 6–10% OTA producers in *A. niger* being the most common frequency [29]–[32].

Previous results indicate that fumonisins and ochratoxins are also produced on other agar media which have a significant amount of nitrogen source and a somewhat lowered water activity caused by addition of NaCl or sucrose [23], [45]. This does not necessarily imply that fumonisins and OTA are produced under industrial conditions, but the results obtained here strongly indicate that many *A. niger* isolates may produce fumonisins in media with rather high amounts of carbohydrate. Such media include those used for citric acid production that contain sucrose or other carbohydrates in high amounts to give the highest possible yields of citric acid or other organic acids [2], [4]. However, other fermentation conditions where *A. niger* is used may also induce production of fumonisins. *A. niger* is listed as a producer of more than 10 enzyme families [6], and none of the substrates used for the fermentations have been examined for their stimulation of fumonisin production. Based on a high production of fumonisins and OTA on CYA with 20% sucrose [23], CYAS and YES [23] agars it is clear that other substrates with high amounts of nitrogen and carbon source including various waste products will likely support toxin production. Fumonisin may be produced given the right physiological conditions like wall growth in a fermentor [46], or in the worst case solid state fermentation, where sporulation often occurs.

Specific attention should be given to the use of fungal mycelium from fermentations as animal feed [47]–[48], where the fumonisin and OTA can be produced by the proposed strain NRRL 337 or any other mycotoxin producing *A. niger* (Table S1).

A large proportion of the strains used for laboratory experiments are derived from a fumonisin producer. For example the strain AB4.1 is derived from N400  =  NRRL 3. These strains have been used in many experiments and a protease deficient strain derived from AB4.1 has been suggested to be used as host for production of human tissue plasminogen activator [19]. It is obviously very important that mycotoxins are not present in such proteins used in human medicine.

It has been suggested that the tricarballylic acid moiety in the fumonsins is derived from the citric acid cycle [1], [49], [50], and a subsequent high flux into citric acid could influence fumonisin production, however no such correlations were found in our experiments. *A. niger* lacks tricarboxylate transporter FUM 11 present in *Fusarium verticillioides*, but *A. niger* has the biochemical ability to produce citric acid and probably recruit the tricarballylic acid via the citric acid biosynthesis. Fumonisin is, however, a small fraction of the output of organic acids on a molar basis.

Since most strains of *A. niger* produce fumonisins (Table 1) it is a possibility to use isolates of other species closely related to *A. niger*, such as *A. tubingensis*, *A. acidus, A. brasiliensis* or *A. vadensis*, none of which produce fumonisins or ochratoxins. As can be seen from the Table S4, these species have also been used for citric acid production. Indeed, several strains have been used by the industry as “*A. niger*”, but have here been shown to represent some of the latter species (Table S4). Among the non-fumonisin producing *A. niger* are NRRL 321, 335, 340, 593 and 595, these were only listed in the original paper on citric acid producing black Aspergilli [42] and have not been used for industrial purposes. NRRL 595 has been used infrequently, however (Table S1, Table S4).

Despite the availability of other fungi in biotechnology as industrial work-horses, it is *Aspergillus niger sensu stricto* that has been used most extensively, also stressed by that fact that it has been genome sequenced three times [1]–[2], [15], [51]. The fact that *A. niger* has been given GRAS status in many industrial applications could be questioned by the fact that most strains of *A. niger* produce fumonisins and some of them in addition OTA, both potentially carcinogenic mycotoxins, on laboratory media. Our findings that some of these industrially used *A. niger* strains can produce these two mycotoxins, at conditions mimicking industrial citric acid production conditions, strongly emphasizes the need for analytical control for securing the absence of mycotoxins in the final industrial products. Currently no validated methods for the analysis of the two toxins in fermentation products have been published, and we highly recommend developing such methods.

Altogether we analyzed all available strains of industrially used *A. niger*, and found a surprisingly high frequency of effective fumonisin producers among them. With the recent development of advanced gene targeting methods in filamentous fungi, site-specific point mutations in essential genes required for production of fumonisin and ochratoxin in *Aspergillus niger* should be used in order to avoid any mycotoxin production in industrial products.

## Supporting Information

Table S1 Table S1 is a list of culture collection strains of *Aspergillus* section *Nigri*, their origin, their alias numbers, and their qualitative fumonisin and ochratoxin A production.
(DOC)

Table S2 Table S2 is a list of black *Aspergillus* strains used in biotechnology that are accessioned in international culture collections and their production of mycotoxin.
(DOC)

Table S3 Table S3 contains strains of *Aspergillus niger* listed according to amounts of fumonisin B_2_, B_4_ and B_6_ produced.
(DOC)

Table S4 Table S4 is a referenced list of citric acid producers, transformation hosts, enzyme, single cell protein, and animal feed producer strains, strains used for biotransforming natural products and strains producing lipids in *Aspergillus* section *Nigri*. This table is followed by 299 supplementary references.
(DOC)

Table S5 Table S5 contains the results of citric acid production, fumonsin, ochratoxin A nad qualitative malformin production on the medium CIT4 by 9 strains of *A. niger*.
(DOC)

Table S6 Table S6 contains the results of citric acid production, fumonsin, ochratoxin A and qualitative malformin production on the medium CIT2 by 9 strains of *A. niger*.
(DOC)

Table S7 Table S7 contains the results on citric acid production, fumonsin, ochratoxin A and qualitative malformin on the medium CIT5 by 9 strains of *A. niger*.
(DOC)
