# Supplementary material for: Fumonisin and Ochratoxin Production in Industrial Aspergillus niger Strains
Source: PLoS One. 2011 Aug 11;6(8):e23496. doi: 10.1371/journal.pone.0023496 (PMC3154942; doi:10.1371/journal.pone.0023496)
Supplement: Table S1 — is a list of culture collection strains of Aspergillus section Nigri, their origin, their alias numbers, and their qualitative fumonisin and ochratoxin A production. (DOC) [file pone.0023496.s001.doc]

**Table S1**. Supplementary material.

Fumonisin and ochratoxin production in industrial *Aspergillus niger* strains.

by

Jens C. Frisvad, Thomas O. Larsen, Ulf Thrane, Martin Meijer, Janos Varga, Robert A. Samson & Kristian Fog Nielsen

**Table S1**. Culture collection strains of *A. niger* and other species in *Aspergillus* section *Nigri*, including known and applied producers of citric acid, gluconic acid, oxalic acid, ascorbic acid, fumaric acid, amylase, lipase, or used as transformations hosts, for biotransformation of extrolites etc. that have been examined in this study. Cultures underlined are the strains actually examined. Each strain number is followed by alias culture collection numbers, which industrial use they are known to have, other scientific names applied to them, original isolation source and country of origin (when known) and a qualitative indication of whether they produce fumonisins and ochratoxins.

*Aspergillus niger* (and *A. awamori*):

ATCC 1015^a^ = IBT 28639 = CBS 113.46 = IBT 27878 = IBT 27877 = IBT 27875 = IBT 27872 = IMI 031821 = NRRL 328 = NRRL 350 = NRRL 511 = NRRL 1278 (citric acid) (+ fumon, - OTA)

NRRL 2270 = IBT 26391 = ATCC 11414 = IBT 28640= IMI 075353 = MZKI A-60 = MZKI A-75 = MZKI A-106 = MZKI A-165 = MZKI A-272 = MZKI A-519 = NRC A-1-233 = VTT D-77050 = Wisconsin 72-4, derived from ATCC 1015 (citric acid, citric acid from apple pomace, aconitic hydrase, carnitine acetyltransferase, citrate lyase, endo-polygalacturonase, 6-phosphofructo-2-kinase, xylanase, **transformation host**) (*A. niger* var. *usamii*) (+ fumon, - OTA)

CBS 102.12^b^ = ATCC 10549 = IBT 19348 = IBT 26343 = IFO 4067 = MUCL 13608 = NRRL 4863 = WB 4863 (amylase, lipase) (*A. niger-altipes***^T^**^,c^) (+ fumon, + OTA)

CBS 103.12b = ATCC 1027 = IBT 16906 = IBT 28139 = IBT 29892 = IFO 4043 = IMI 016148 = LSBH Ac42 = MZKI A-76 = NCTC 3774 = NRRL 348 = QM 326 = Thom 3534B = WB 348 (amylase, lipase) (*A. cinnamomeus***^T^**, *A. niger* var. *niger* f*. hennebergii*) (+ fumon, - OTA)

CBS 121.28 = IFO 4031 = IMI 104687 = NRRL 4784 = WB 4784 (awamori-koji, amylase, lipase) (Japan) (*A. foetidus*, *A. aureus* **^T^**) (+ fumon, - OTA)

CBS 109.30^b^ (Germany) (citric acid, oxalic acid) (+ fumon, + OTA)

CBS 110.30 (Germany) (citric acid, oxalic acid, amylase, lipase) (*A. niger* var. *nanus*) (+ fumon, + OTA)

CBS 111.30^b^ (Germany) (citric acid) (+ fumon, - OTA)

CBS 112.30 (Germany) (citric acid) (ex *Prunus insititia*) (+ fumon, weak, - OTA)

CBS 113.30^b^ (Germany) (oxalic acid, amylase, lipase) (+ fumon, - OTA)

CBS 112.32 (Japan) (+ fumon, weak, - OTA)

CBS 113.33 = ATCC 10548 = IBT 16911 = NRRL 4864 = WB 4864 (Germany) (amylase, lipase) (*A. niger* mut. fusca, *A. foetidus*, *A. awamori*) (+ fumon, - OTA)

CBS 118.36 = IBT 28105 (ex chemical, USA) (amylase, lipase) (*A. niger*, *A. phoenicis*) (+ fumon, - OTA)

CBS 108.47 (ascorbic acid, citric acid) (+ fumon, - OTA)

CBS 124.48 (glucose oxidase) (+ fumon, - OTA)

CBS 112.52 = Ciferri 936 = IBT 4967 = NRRL 4840 = WB 4840 (Japan) (*A. aureus* var. *acidus*, *A. niger* var. *awamori*) (+ fumon, + OTA)

CBS 113.52 = Ciferri 746 = NRRL 4841 = WB 4841 (amylase, lipase) (*A. niger*, *A. aureus* var. *brevius*) (+ fumon, + OTA)

CBS 116.52 = IBT 16908 (*A. awamori* var. *fumeus*) (+ fumon, - OTA)

CBS 117.52 = Ciferri 931 = NRRL 4844 = WB 4844 (ex koji) (amylase, lipase) (*A. awamori* var. *fuscus***^T^,** *A. niger* var. *intermedius*) (+ fumon, + OTA)

CBS 131.52 = IBT 3255 = IBT 5772 = ATCC 6275 = CBS 113.50 = IBT 28096 = CBS 769.97 = CECT 2807 = DSM 1957 = IFO 6341 = IFO 6341 = IMI 045551 = MUCL 19002 = MZKI A-98 = MZKI A-148 = NRRL 334 = NRRL 613 = QM 324 = QM 458 = C. Thom 4247-215 = USDA 215-4247 = VTT D-81078 = WB 334 (ex leather) (citric acid, degradation of apple distillery waste, used for fungus resistance testing; amylase, cellulase, lipase) (*A. niger* var. *nanus*) (+ fumon, - OTA)

CBS 122.55^b^ (ex ear of man, Switzerland) (+ fumon, - OTA)

CBS 262.65 = IBT 28140 (+ fumon, - OTA)

CBS 263.65 = IMI 266386 (amylase, lipase) (+ fumon, weak, - OTA)

CBS 555.65 = ATCC 16882 = IBT 4385 = IBT 29901 = IHEM 3710 = IMI 091881 = LSBH Ac3 = NRRL 364 = QM 8887 = Thom 142 = WB 364 (citric acid, oxalic acid, amylase, lipase, polygalacturonase) (*A. ficuum*, *A. niger* var. *usamii*) (+ fumon, - OTA)

CBS 563.65 = ATCC 16878 = CBS 114.49 = IBT 5957 = MUCL 13601 = NRRL 341 = C. Thom 5135.17 = WB 341 (*A. foetidus***^T^**, *A. aureus, A. niger* var. *awamori*) (+ fumon, weak, - OTA)

CBS 103.66 = IMI 266384 (+ fumon, weak, - OTA)

CBS 623.78 = IMI 313770 = NRRL 367 = C. Thom 4640.480 (France) (+ fumon, - OTA)

CBS 630.78 = ATCC 52007 = IBT 28097 = IMI 313484 = MZKI A-136 = NRRL 1956 = QM 329 = WB 1956 (army equipment, South Pacific Island) (glucosidase) (*A. foetidus*, *A. phoenicis*) (+ fumon, - OTA)

CBS 107.80^b^ (mutant of CBS 113.30) (Germany) (amylase, lipase) (+ fumon, - OTA)

CBS 108.80^b^ (mutant of CBS 113.30) (Germany) (amylase, lipase) (+ fumon, weak, - OTA)

CBS 117.80^b^ = IBT 4983 = IFO 4308 = NRRL 4886 = WB 4886 (pectinase, rhamnogalacturonase) (*A. niger* var. *niger* f. *hennebergii*, *A. kawachii***^T^**) (+ fumon, - OTA)

IMI 016141 = IBT 23721 (= CBS 307.80?) = LSBH Ac103 = NCTC 3892 (fumaric acid) (+ fumon, + OTA)

CBS 101698 = IBT 21853 (ex coffee, Kenya) (+ fumon, - OTA)

CBS 101703 = IFO 4397 = IBT 19350 (+ fumon, + OTA)

CBS 101705 = IBT 28144 (ex indoor air, Canada) (+ fumon, + OTA)

CBS 101706 = CECT 20157 = IBT 20181 = IMI 367497 (ex soya bean) (+ fumon, + OTA)

CBS 101707 = CECT 20156 = IBT 20182 = IMI 367496 (+ fumon, weak, OTA precursor)

CBS 117785^b^ = IBT 28090 (ex date, Marocco) (+ fumon, - OTA)

CBS 118725^b^ = IBT 28098 (ex *Hyacinthus* bulb, Netherlands) (+ fumon, - OTA)

IFO 4122 = IBT 19349 = CBS 101708 (*A. awamori* var. *fumeus*) (+ fumon, + OTA)

IFO 6082 = CBS 101700 = FAT 407 = IBT 19345 (*A. usamii* mut. shiro-usamii) (+ fumon, + OTA)

IFO 8875 = CBS 101702 = IBT 19351 (*A. usamii*) (+ fumon, precursor to OTA)

ITEM 7097^b^ = IBT 28086 (+ fumon, - OTA)

NRRL 3^a^ = ATCC 9029 = BCRC 32720 = CBS 120.49 = CECT 2088 = DSM 2466 = IBT 23539 = IBT 29897 = IMI 041876 = MUCL 30480 = MZKI A-158 = N400 =NRRL 566 = VKM F-3747 = VTT D-85240 = WB 3 = WB 566 (USA) (gluconic acid, citric acid, glucono-δ-lactone, glucose oxidase, amylase, lipase; nigeran, treatment of pulp mill wastes, lyncomycin sulphoxide, **transformation host**) (+ fumon, - OTA)

N402 = IBT 28472 (derived from N400 = NRRL 3) (+ fumon, - OTA)

AB4.1 = ATCC 62590 (derived from N400 = NRRL 3) (+ fumon, - OTA)

FGSC A742 (derived from N400 = NRRL 3) (+ fumon, - OTA)

NRRL 326**^T^** = ATCC 16888 = CBS 554.65 = IBT 27876 = IFO 33023 = IHEM 3415 = IMI 050566ii = JCM 10254 = C. Thom 2766 = WB 326 (ex tannin acid fermentation) (citric acid, gluconic acid, tannic acid fermentation, glucose oxidase, β-glucosidase, kynureninase-type enzymes; treatment of pulp-mill wastes, chemical transformation of costunolide, acetanilide and pergolide; production of pseudonigerone) (+ fumon, - OTA)

NRRL 330 = ATCC 10864 (= CBS 122.49?) = IFO 6661 = IBT 27874 = IMI 060286 (ex galls, China) (acetylesterase, acetyl-xylan esterase, α-amylase, glucoamylase, maltase, monoamine oxidase; ethanol from potato starch; **transformation host**) (+ fumon, - OTA)

NRRL 337 = ATCC 10254 = Biourge 739 = CBS 126.48 = CBS 618.78 = CCRC 30206 = DSM 734 = IBT 3277 = IBT 25294 = IBT 23679 = IBT 23680 = IBT 23681 = IFO 6428 = IMI 015954 = IMI 041871 = MZKI A-110 = C. Thom 4668.4 = WB 337 (citric acid, amylase, amyloglucosidase, lipase, maltase, monooxygenase, fungal protein from food waste for animal feed) (*A. foetidus*, *A. citricus*) (+ fumon, + OTA)

NRRL 357 = ATCC 1035 = IBT 29893 = IMI 015953 = Thom 4828.8 = WB 357 (*A. foetidus* var*. pallidus*, *A. niger, A. luchuensis*) (+ fumon, - OTA)

NRRL 362^b^ = IBT 29882 (+ fumon, - OTA)

NRRL 372^b^ = ATCC 12845 = IBT 29890 = IMI 084304 = NRRL 619 = QM 1999 = C. Thom 5087 = WB 372 (*A. ficuum*) (citric acid from crude molasses) (+ fumon, - OTA)

NRRL 567^b^ = ATCC 12846 = IBT 26387 = IBT 26437 = MZKI A-62 = QM 6742 = WB 567 (citric acid, citric acid from apple pomace, citric acid from grape pomace, gluconic acid) (+ fumon, - OTA)

NRRL 599 = ATCC 9142 = ATCC 74337 = CCM F-320 = CECT 2090 = DSM 821 = IBT

26389 = IMI 041874 = MZKI A-61 = WB 599 (citric acid) (+ fumon, - OTA)

NRRL 604^b^ = IBT 29895 (citric acid) (+ fumon, - OTA)

NRRL 611 = IBT 29887 (citric acid) (+ fumon, - OTA)

NRRL 612 = ATCC 7983 = IBT 29885 = 45-Porges (citric acid) (+ fumon, - OTA)

NRRL 615 = IBT 29884 (citric acid) (+ fumon, - OTA)

NRRL 1213 = ATCC 7797 = Dow Chemical Co. 29 = IBT 29889 (fungus resistance of oil paintings) (+ fumon, - OTA)

NRRL 2001^b^ = ATCC 13794 = IBT 26392 = IMI 051433 = MZKI A-77 = QM 6863 = WB 2001 (originally as *A. wentii*) (citric acid, citric acid from apple pomace) (+ fumon, - OTA)

NRRL 3112 = ATCC 22342 = CBS 115988 = IBT 23540 (α-amylase, amyloglucosidase, aminopeptidase; **transformation host**: transgenic production of chymosin B) (originally identified as *A. awamori*) (+ fumon,+ OTA)

NRRL 3122 = ATCC 22343 = CBS 115989 = IBT 23538 (α-amylase, amyloglucosidase, feed from peel waste) (+ fumon, + OTA)

CBS 513.88^a^ = IBT 29270 = IBT 29709, derived from NRRL 3122 (+ fumon, + OTA)

NRRL 6408^b^ = IBT 29888 (ex corn, USA) (+ fumon, - OTA)

*Aspergillus niger*, fumonisin not detected:

CBS 118.35 = CECT 2801 = IBT 3662 = NRRL 4883 = QM 9704 = WB 4883 (*A. hennebergii*^T^) (- fumon, - OTA)

CBS 127.48 = ATCC 44733 = Biourge 746 = NRRL 4869 = WB 4869 (*A. niger,* *A. awamori*, *A. pseudo-citricus***^T^**) (degradation of raffinose, production of α-galactosidase, invertase) (-fumon, + OTA)

CBS 114.50 (yellow mutant of CBS 113.50) (- fumon, - OTA)

CBS 139.52 = ATCC 11964 = ATCC 14331 = IAM 2185 = IBT 3256 = IBT 5790 = IBT 28099 = IFO 4388 = NRRL 4760 = QM 8164 = R. 0635 = WB 4760 (ex kuro-koji, Japan) (acid protease, acid carboxypeptidase) (*A. awamori*, *A. usamii***^T^**, *A. niger* var. *usamii*) (- fumon, + OTA)

CBS 139.54^b^ (ex *Welwitschia mirabilis*, Namibia) (*A. welwitschiae, A. niger*) (- fumon, - OTA)

CBS 121.55^b^ (ex otomycosis, man, Switzerland) (*A. niger* var. *usamii*) (- fumon, - OTA)

CBS 557.65^b^ = ATCC 16877 = IMI 211394 = NRRL 4948 = WB 4948 (*A. awamori*^N^**^T^**) (- fumon, - OTA)

CBS 617.78^b^ = IBT 3277 = NRRL 4571 (ex soft rot of *Dracaena* sp.) (*A. ficuum*) (- fumon)

CBS 101699^b^ = IBT 6461 (ex food) (- fumon, + OTA)

CBS 101701 = IBT 19347 = IFO 8877 (*A. usamii*) (- fumon, + OTA)

CBS 101704 = IBT 19346 = IFO 8876 (*A. usamii*) (- fumon, + OTA)

NRRL 320^b^ = CBS 616.78 = IBT 3756 = IBT 28861= IMI 358439 = QM 8913 = C. Thom 4640.412 = WB 320 (Brazil) (*A. ficuum, Sterigmatocystis pseudocarbonaria*) (- fumon, - OTA)

NRRL 321^b^ = IBT 29900 (citric acid) (*A. niger*) (- fumon, - OTA)

NRRL 335^b^ = IBT 29896 (citric acid) (*A. niger*) (- fumon, - OTA)

NRRL 340^b^ = IBT 29879 (citric acid) (*A. niger*) (- fumon, - OTA)

NRRL 356 = IBT 29903 = ATCC 10061 = IMI 015953 = NCTC 1017 = QM 334 = Thom 4291.3 (*A. awamori*, *A. luchuensis*) (- fumon, - OTA)

NRRL 363 = ATCC 10698 = CBS 126.49 = IAM 2190 = IBT 3617 = IBT 5794 = IBT 29899 = IFO 4034 = IFO 6648 = C. Thom 5135.16 (*A. awamori*, *A. batatae*, *A. phoenicis*, *A. ficuum*, *A. niger* var. *phoenicis*) (- fumon, + OTA)

NRRL 593^b^ = AJ Moyer strain Cardozo 477 = ATCC 12847 = IBT 29891 = MZKI A-135 (*A. phoenicis*, *A. tubingensis*) (citric acid) (- fumon, - OTA)

NRRL 595^b^ = IBT 29894 (super citric acid producer; citric acid from crude date syrup; heavy metal treatment, microbial transformation of diosgenin) (- fumon, - OTA)

NRRL 4857 = CBS 121.48 = Mosseray 699 = WB 4857 (*A. longobasidia*, *A. niger* var. *awamori*) (- fumon, - OTA)

*Aspergillus acidus*:

CBS 105.47 = A2 = IBT 3412 (Switzerland) (citric acid, gluconic acid, riboflavin, amylase, lipase) (*A. niger* var. *nanus*) (- fumon, - OTA)

CBS 107.47 (Basel, Schwitzerland), (amylase, lipase, citric acid, ascorbic acid) (*A. niger*), (- fumon, - OTA)

CBS 117.32 = IBT 3252 (amylase, lipase) (*A. ficuum*, *A. niger* var. *intermedius*) (- fumon, - OTA)

CBS 114.52 = Ciferri 946 = IBT 4935 = NRRL 4842 = WB 4842 (amylase, lipase) (*A. foetidus, A. aureus* var. *pallidus*) (- fumon, - OTA)

CBS 115.52 = ATCC 11358 = IBT 4976 = Sakaguchi K. 6615 (ex kuro-koji for sweet potato destilled wine) (amylase, lipase, **transformation host**) (*A. foetidus*, *A. awamori*) (- fumon, - OTA)

CBS 119.52 = Ciferri 939 = IBT 23470 = NRRL 4846 = WB 4846 (*A. awamori, A. awamori* var. *piceus*) (- fumon, - OTA)

CBS 126.52 = Ciferri 924 = IBT 4981 = IFO 4125 = NRRL 4860 = WB 4860 (*A. miyakoensis, A. foetidus*) (amylase, lipase) (- fumon, - OTA)

CBS 553.65 = ATCC 16880 = IBT 28093 = IBT 28612 = IMI 235599 = NRRL 5121 = WB 5121 (ex soil, Costa Rica) (*A. niger* var. *usamii*) (- fumon, - OTA)

CBS 565.65 = ATCC 16884 = IBT 3644 = IBT 24701 = IFO 4123 = IMI 175963 = NRRL 4797 = WB 4797 (*A. foetidus*, *A. foetidus* var. *pallidus***^T^**) (Japan) (- fumon, - OTA)

IMI 104688 = ATCC 16874 = CBS 564.65 = IBT 3410 = IFO 4121 = IMI 104688 = NRRL 4796 = WB 4796 (*A. foetidus*, *A. foetidus* var. *acidus***^T^**) (Japan) (- fumon, - OTA)

KACC 41731 = IBT 27923 = KCCM 80006 = KCTC 18075P (*Aspergillus coreanus***^T^**) (ex nuruk, Korea) (- fumon, - OTA)

KACC 41733 = IBT 27924 (*Aspergillus coreanus*) (ex nuruk, Korea) (- fumon, - OTA)

NRRL 2322 = NRRL 2354 = ATCC 10577 = Clement A-1-215 = DSM 823 = IAM 3010 = IBT 26390 = IBT 28862 = IMI 027809 = MZKI A-67 = MZKI A-73 = MKZI A-100 = NCTC 7193 = QM 6906 (citric acid from beet molasses and brewery wastes, gluconic acid, L-malic acid) (*A. niger*) (- fumon, - OTA)

NRRL 4750 = CBS 128.52 = IBT 29904 = QM 8183 = WB 4750 (*A. foetidus* var. *acidus*, *A. phoenicis*) (Japan) (- fumon, - OTA)

IBT 4570, IBT 4602, IBT 4935, IBT 16906, IBT 24825, IBT 28093, IBT 28095, IBT 29186, IBT 29214, IBT 29217, IBT 29220, IBT 29221 + 15 further strains (all – fumon, - OTA)

*Aspergillus aculeatinus*:

CBS 121060**^T^** = IBT 29077 (Arabica green coffee, Thailand) (- fumon, - OTA)

CBS 121061 (Arabica green coffee, Thailand) (- fumon, - OTA)

CBS 121062 (Arabica green coffee, Thailand) (- fumon, - OTA)

CBS 121871 (Arabica green coffee, Thailand) (- fumon, - OTA)

CBS 121872 (Arabica green coffee, Thailand) (- fumon, - OTA)

CBS 121873 (Arabica green coffee, Thailand) (- fumon, - OTA)

CBS 121874 (Arabica green coffee, Thailand) (- fumon, - OTA)

CBS 121875 = IBT 29118 (Arabica green coffee, Thailand) (- fumon, - OTA)

6 more strains from green Arabica coffee, Thailand (all – fumon, - OTA)

*Aspergillus aculeatus*

CBS 122.35 = IBT 29560 = NRRL 4880 = WB 4880 (*A. violaceofuscus***^T^**) (- fumon, - OTA)

CBS 101.43 = DSM 2344 = IBT 3411 = IBT 3689 (ex wood of *Santalum* sp.) (amylase, lipase) (- fumon, - OTA)

CBS 119.49 = IBT 4580 (ex *Lactuca sativa*, Indonesia) (- fumon, - OTA)

CBS 172.66**^T^** = ATCC 16872 = IBT 3244 = IMI 211388 = NRRL 5094 = WB 5094 (ex tropical soil) (- fumon, - OTA)

CBS 186.67 = MUCL 6225 (ex litter, Zaire) (- fumon, - OTA)

CBS 610.78 = ATCC 1034 = IMI 119894 = NRRL 358 = C. Thom 4030.3 = WB 358 (ex tropical soil) (- fumon, - OTA)

CBS 611.78 = NRRL 5118 (ex tropical soil) (- fumon, - OTA)

CBS 621.78 = IBT 3249 = IMI 312981 = NRRL 4912 = WB 4912 (*A. brunneoviolaceus***^T^**) (- fumon, - OTA)

CBS 115.80 = IBT 3243 = IFO 5330 (lipase) (*A. yezoensis***^T^**) (- fumon, - OTA)

CBS 116.80 = IBT 4972 = IMI 083356 (ex palm oil, Nigeria) (- fumon, - OTA)

CBS 308.80 = IMI 054395 ( ex soil, Nigeria) (- fumon, - OTA)

CBS 721.96 (ex branch, Papua-New Guinea) (- fumon, - OTA)

IBT 29222 (ex cocoa, Brazil) (- fumon, - OTA)

IBT 29275 (Brazil) (- fumon, - OTA)

*Aspergillus brasiliensis*:

ATCC 9642 = AMP 26 = ATHUM 2856 = CBS 246.65 = CECT 2700 = DSM 63263 = IFO 6342 = IHEM 3797 = IBT 13099 = IBT 28083 = IBT 29883 = IMI 091855iii = IMI 149007 = LCP 74.2280 = MUCL 19001 = MZKI A-164 = NDRC SN26 = NRRL A-5243 = NRRL 3536 = OECD 14 = QM 386 = VKM F-1119 = Weston SN-26 (ex radio equipment, Sydney) (amylase, isopullulanase, lipase, used for fungus resistance testing of plastics and wood preservatives, biotransformation (*A. niger* var. *usamii*)) (- fumon, - OTA)

CBS 626.66 = IBT 28114 = IMI 114927 (France) (- fumon, - OTA)

CBS 733.88 = ATCC 16404 = B39906 = B42936 = CECT 2574 = DSM 1387 = DSM 1988 = IBT 28084 = IFO 9455 = IHEM 2311 = IHEM 3766 = IHEM 3794 = IMI 149007 = IP 1431.83 = MUCL 29039 = MUCL 30113 = NCPF 2275 (ex *Vaccinium* s/g *Cyanococcus*, North Carolina, USA) (antimicrobial preservative testing, **transformation host**) (- fumon, - OTA)

CBS 101740**^T^** = IBT 21946 = IBT 26433 = IMI 381727 = JHC 614 (ex soil, Brazil) (- fumon, - OTA)

CBS 116970 = IBT 28085 (ex soil, Netherlands) (- fumon, - OTA)

ITEM 4540 = IBT 28176 (grapes, Portugal) (- fumon, - OTA)

ITEM 4544 = IBT 28177 (grapes, Portugal (- fumon, - OTA)

ITEM 6139 = IBT 28180 (grapes, Portugal) (- fumon, - OTA)

JHC 603 = IBT 26434 = IBT 28178 (soil, Brazil) (- fumon, - OTA)

JHC 605 = IBT 28179 (soil, Brazil) (- fumon, - OTA)

JHC 606 = IBT 28175 (soil, Brazil) (- fumon, - OTA)

6 further isolater (all – fumon, - OTA)

*Aspergillus carbonarius*

CBS 111.26**^T^** = ATCC 1025 = ATHUM 2854 = CBS 556.65 = IBT 3245 = IBT 3687 = IMI 016136ii = LSBH Ac11 = MUCL 13583 = NCTC 1325 = NRRL 369 = NRRL 1987 = QM 331 = C. Thom 40301 = WB 369 (ex paper) (amylase, lipase) (- fumon, + OTA)

CBS 114.29 = IBT 4591 = WB 4849 (*Sterigmatocystis acini-uvae*) (- fumon, + OTA)

CBS 114.46 = ATCC 6276 = CBS 112.80 = CECT 2086 = IBT 3246 = IBT 5712 = IMI 041873 = NRRL 368 = USDA 244-4707.878 = C. Thom 4707.878 = WB 368 (citric acid) (- fumon, + OTA)

CBS 110.49 (ex air, Indonesia) (- fumon, + OTA)

IBT 29171 (ex cocoa, Brazil) (- fumon, + OTA)

IBT 29172 (ex cocoa, Braxil) (- fumon, + OTA)

IBT 29184 (ex cocoa, Brazil) (- fumon, + OTA)

NRRL 67 = ATCC 8740 = CBS 420.64 = DSM 872 = IBT 27879 = IBT 28858 = IMI 041875 = Moyer 67 = MUCL 30479 = NRRL 605 = NRRL 1737 = QM 330 = C. Thom 4707.878 = WB 67 = Wis. 62 (“old series”) (citric acid, gluconic acid, amylase, lipase) (- fumon, + OTA)

CBS 113.80 = IBT 4355 = IMI 132655 (ex *Theobroma cacao*, Nigeria) (- fumon, + OTA)

CBS 111.80 = IBT 13987 = IMI 138280 (South Africa) (amylase, lipase) (- fumon, + OTA)

IBT 21854 (ex coffee, India) (- fumon, + OTA)

IBT 22904 (ex grape, Greece) (- fumon, + OTA)

18 further strains from coffee (Thailand) (- fumon, + OTA)

*Aspergillus costaricaensis*:

CBS 115574**^T^** = IBT 23401 (ex soil, Costa Rica) (- fumon, - OTA)

*Aspergillus ellipticus*

CBS 677.79 = IBT 12963 (ex soil, Costa Rica) (- fumon, - OTA)

CBS 707.79**^T^** = ATCC 16876 = CBS 482.65 = IBT 3278 = IBT 3678 = IBT 13962 = IMI 172283 = WB 5120 (ex soil Costa Rica) (- fumon, - OTA)

*Aspergillus heteromorphus*

CBS 117.55**^T^** = ATCC 12064 = IBT 3247 = IBT 3622 = IBT 5717 = IBT 13961 = IMI 172288 = QM 6959 = WB 4747 (culture contaminant, Brazil) (- fumon, - OTA)

*Aspergillus homomorphus*

CBS 101889**^T^** = CMPG 1276 = IBT 21893 (ex sand, Israel) (- fumon, - OTA)

IBT 21894 = CMPG 1277 = CBS 101888 (ex sand, Israel) (- fumon, - OTA)

*Aspergillus ibericus*

CBS 121593**^T^** = IBT 26612 = ITEM 4776 = IMI 391429 (ex grapes, Portugal) (- fumon, - OTA)

IBT 29169 (cocoa, Brazil) (- fumon, - OTA)

IBT 29170 (cocoa, Brazil) (- fumon, - OTA)

*Aspergillus japonicus*

CBS 114.51**^T^** = IBT 3248 = IBT 5718 (lipase) (- fumon, - OTA)

CBS 114.34 = IBT 5031 = IBT 22022 = IFO 4062 = IMI 312982 = NRRL 4786 = WB 4786 (ex skin of man, Japan) (- fumon, - OTA)

CBS 113.48 = Biourge 687 = IBT 3614 = IMI 016135ii = IMI 312983 = LSHB Ac44 = MUCL 13578 = NCTC 3792 = NRRL 4839 = C. Thom 4724.44 = WB 4839 (*A. atroviolaceus***^T^**) (- fumon, - OTA)

IBT 28231 (ex soil, Australia) (- fumon, - OTA)

*Aspergillus lacticoffeatus* (filed under *Aspergillus niger*)

CBS 101883**^T^** = IBT 22031 (ex green coffee beans, Indonesia) (+ fumon, + OTA)

CBS 101885 = IBT 22029 (ex green coffee beans, Venezuela) (- fumon, + OTA)

CBS 101884 = IBT 22030 (ex green coffee beans, Venezuela) (- fumon, + OTA)

CBS 101886 = IBT 22032 (ex green coffee beans, Indonesia) (- fumon, + OTA)

*Aspergillus piperis*

CBS 112811**^T^** = IBT 24630 (ex black pepper, Denmark) (- fumon, - OTA)

*Aspergillus sclerotiicarbonarius*

CBS 101057**^T^** = IBT 28362 (ex green coffee beans, Thailand) (- fumon, - OTA)

CBS 101056 = IBT 28363 (ex green coffee beans, Thailand) (- fumon, - OTA)

4 more strains form green coffee beans, Thailand (- fumon, - OTA)

*Aspergillus sclerotioniger*

CBS 115572**^T^** = IBT 22905 (ex green coffee bean, India) (- fumon, + OTA)

*A. tubingensis*:

CBS 115.29**^T^** = CBS 559.65 = ATCC 10550 = IBT 4323 = IMI 172296 = NRRL 4866 = WB 4866 (*A. niger* var. *intermedius, A. niger* f. *tübingen*) (- fumon, - OTA)

CBS 117.32 = IBT 3252 (*A. niger* var. *intermedius*) (amylase, lipase) (- fumon, - OTA)

CBS 111.34 (*A. awamori*) (Japan) (- fumon, - OTA)

CBS 115.34 = IBT 4579 = IFO 4034 = IMI 313486 = NRRL 4785 = WB 4785 (*A. ficuum*, *A. batatae*) (amylase, lipase) (- fumon, - OTA)

CBS 116.36 = ATCC 10553 = IBT 28137 (Russia, treated with radium) (- fumon, - OTA)

CBS 114.37 = IAM 2185 = IBT 4944 = IBT 29898 = IMI 313493 = NRRL 4856 = WB 4856 (*A. japonicus*, *A. luchuensis* var. *rubeolus*, *A phoenicis, A. niger* var. *usamii*) (amylase, lipase) (kuro-koji) (- fumon, - OTA)

CBS 124.38 (*A. niger*) (amylase, lipase, citric acid) (- fumon, - OTA)

CBS 114.39 = MZKI A-63 (citric acid) (- fumon, - OTA)

CBS 102.40 = IMI 094145 (produces nigeran) (- fumon, - OTA)

CBS 110.42 (*A. niger*) (amylase, lipase, β-xylanase, β-xylosidase, citric acid) (- fumon, - OTA)

CBS 107.47 (ascorbic acid, citric acid, amylase, lipase) (Schwitzerland) (- fumon, - OTA)

CBS 109.47 (ascorbic acid, citric acid) (- fumon, - OTA)

CBS 115.48 = CBS 558.65 = ATCC 16879 = Biourge No. 649a = IBT 3257 = IBT 3613 = IBT 29881 = IMI 211396 = MUCL 15630 = MUCL 13592 = NRRL 4851 = QM 8918 = WB 4851 (*A. eliator*^T^, *A. pulverulentus*, *A. phoenicis, A. niger* var. *phoenicis* f. *pulverulentus*) (amylase, lipase) (- fumon, - OTA)

CBS 117.48 = Biourge 734 = IBT 4351 (*A. fuliginosus*, *A. niger* var. *nanus*) (- fumon, - OTA)

CBS 121.48 = NRRL 4857 = WB 4857 (*A. longobasdidia*, *A. awamori*) (amylase, lipase) (-fumon, - OTA)

CBS 123.48 = ATCC 10575 = ATCC 16019 = DSM 2143 = IMI 017454 = MUCL 19004 = MUCL 19005 = QM 8404 (*A. niger*) (amylase, lipase) (- fumon, - OTA)

CBS 125.48 = P. Biourge 72 (*A. niger*) (- fumon, - OTA)

CBS 128.48 = Biourge 723 = IBT 4356 = IMI 313491 = NRRL 4870 = WB 4870 (*A. pseudo-niger***^T^**, *A. awamori*) (amylase, lipase) (- fumon, - OTA)

CBS 134.48 (lipase) (- fumon, - OTA)

CBS 135.48 (amylase, lipase) (- fumon, - OTA)

CBS 123.49 (*A. niger*) (Germany) (- fumon, - OTA)

CBS 127.49 = IBT 4353 = IMI 313489 = NRRL 4871 = WB 4871 (ex *Coffea arabica*) (*A. pulchellus*) (- fumon, - OTA)

CBS 115.50 = FA 308 (mutant of CBS 113.50 = USDA TC 4247) (*A. niger* var. *nanus***)** (- fumon, - OTA)

CBS 130.52 = ATCC 11394 = IBT 4357 = IMI 076837 = Wisc. 72-2 (culture contaminant) (*A. niger* var. *intermedius*, *A. niger*) (- fumon, - OTA)

CBS 137.52 = ATCC 11363 = IAM 2190 = IBT 4358 = IMI 214827 = K. 3931 = NRRL 4758 = QM 8162 = WB 4758 (*A. saitoi* var. *kagoshimaensis, A. niger* var. *phoenicis*) (amylase, lipase) (- fumon, - OTA)

CBS 134.54 (mutant B50) (- fumon, - OTA)

CBS 136.54 (mutant A4, *A. niger* var. *niger* f. *cinnamomeus, A. niger* ) (- fumon, - OTA)

CBS 137.54 (*A. phoenicis*) (Limoges, France) (- fumon, - OTA)

CBS 107.55 = ATCC 12074 = IBT 4384 = IBT 28115 = NRRL 4740 = WB 4740 (ex skin lesion of man, Brazil) (*A. awamori* var. *hominis,* *A. phoenicis, A. niger* var. *intermedius*) (- fumon, - OTA)

CBS 109.80 (amylase, lipase), (*A. niger* var *niger* f. *hennebergii*, mutant of CBS 114.37))(- fumon, - OTA)

CBS 205.80 = IFO 428 (*A. niger* var. *intermedius*)(- fumon, - OTA)

CBS 306.80 = IBT 28108 = IMI 091886 (ex *Capsicum* sp., Spain) (*A. niger* var. *nanus*) (- fumon, - OTA)

CBS 552.85 = IBT 28094 (ex rhizosphere grass, Lucknow, India) (- fumon, - OTA)

CBS 643.92 = IBT 3567 (- fumon, - OTA)

CBS 242.93 = IBT 28088 (- fumon, - OTA)

CBS 102398 (ex pectin, Netherlands) (- fumon, - OTA)

CBS 116417 = IBT 28111 (- fumon, - OTA)

NRRL 322 = ATCC 1004 = CBS 104.57 = IBT 28857 = IBT 29880 = IMI 031276 = IMI 050565ii = LSBH Ac2 = LSBH Ac13 = MUCL 30481 = NCTC 594 = NCTC 3902 = QM 323 = C. Thom 111 = USDA 215-111 = VKM F-2093 = WB 322 (*A. niger, A. niger* var. *usamii*) (notatin, amylase, lipase, glucose oxidase) (- fumon, - OTA)

NRRL 344 = ATCC 60761 = IBT 29959 = K.A. Seifert WFPL 207B = Thom 5292 = UBC 872 (ex cotton boll, Texas, USA) (evaluation of wood preservative chemicals) (as *A. niger*) (- fumon, - OTA)

NRRL 361 = ATCC 1040 = CBS 122.28 = IFO 4091 = IMI 016269 = IMI 091895 = LSHB Ac37 = NCTC 3781 = QM 327 = Thom 3534c = WB 361 (amylase, lipase) (*A. schiemannii***^T^**, *A. fuscus, A. niger* var*. niger* f. *hennebergii*) (Germany) (- fumon, - OTA)

NRRL 365 = CBS 629.78 = IBT 29902 = IMI 313487 = IMI 358443 = Thom 4640.484 (*A. phoenicis*) (- fumon, - OTA)

NRRL 2295 = ATCC 10581 = CBS 121.49 = IBT 4581 = IBT 27873 = IMI 031283 = Mulder strain = Wageningen 1949 (amylase, lipase; assay of Mg, Cu, Zn and Mo in soil; transformation of pergolide to pergolide sulfoxid) (*A. niger* var. *nanus*) (- fumon, - OTA)

NRRL 3135 = ATCC 66876 = IBT 26388 = IBT 28871 = SRRC 235 (*A. ficuum*) (acid phosphatases, 3-phytase) (- fumon, - OTA)

NRRL 4700 = ATCC 76608 = CBS 161.79 = IBT 4560 = IBT 23488 (ex soil, India) (- fumon, - OTA)

NRRL 4757 = ATCC 11362 = CBS 136.52 = CBS 552.65 = H 217 = IAM 2209 = IBT 3253 = IBT 29886 = IMI 211395 = R-6711 = WB 4757 (ex kuro-koji, Japan) (*A. acidus*, *A. niger* var. *nanus*, *A. phoenicis*, *A. saitoi***^T^**) (- fumon, - OTA)

34 further strains: (- fumon, - OTA)

*Aspergillus uvarum*

CBS 121591**^T^** = IBT 26606 = IMI 388523 = ITEM 4834 (ex grape, Italy) (- fumon, - OTA)

CBS 121590 = IBT 27814 = IMI 388545 = ITEM 4856 (ex grape, Italy) (- fumon, - OTA)

IBT 27815 = ITEM 5024 (ex grape, Italy) (- fumon, - OTA)

IBT 27816 = ITEM 5063 (ex grape, Italy) (- fumon, - OTA)

IBT 27838 = ITEM 5056 (ex grape, Italy) (- fumon, - OTA)

*Aspergillus vadensis*

CBS 113365**^T^** = CBS 102787 = IBT 24658 = IMI 142717 (ex air, Egypt) (- fumon, - OTA)

^a^ Full genome sequenced strains

^b^ These strains were sequenced using β-tubulin in this study and found to belong to the phylospecies *Aspergillus awamori*.

^c^ **T** means culture ex type
