# Supplementary material for: Fumonisin and Ochratoxin Production in Industrial Aspergillus niger Strains
Source: PLoS One. 2011 Aug 11;6(8):e23496. doi: 10.1371/journal.pone.0023496 (PMC3154942; doi:10.1371/journal.pone.0023496)
Supplement: Table S2 — is a list of black Aspergillus strains used in biotechnology that are accessioned in international culture collections and their production of mycotoxin. (DOC) [file pone.0023496.s002.doc]

**Table S2**. Supplementary material.

Fumonisin and ochratoxin production in industrial *Aspergillus niger* strains.

by

Jens C. Frisvad, Thomas O. Larsen, Ulf Thrane, Martin Meijer, Janos Varga, Robert A. Samson & Kristian Fog Nielsen

**Table S2**. Black *Aspergillus* strains used in biotechnology that are accessioned in international culture collections and their production of mycotoxins on CYAS agar. The number of citations for industrial applications are also listed.

| Fungus |  | Mycotoxin  production |  | Industrial  applications |  |  |  |
| --- | --- | --- | --- | --- | --- | --- | --- |
| Species | Strain | Fumonisin  B_2_ production | Ochratoxin  A production | Citric and other acids | Hetero-  logous  protein | Enzyme production | Bio-  trans-  formation |
| *A. acidus* (**N**)^a^ | CBS  115.52 | - | - |  | 8^b^ | 1 |  |
|  | CBS 126.52 | - | - | 1 |  | 1 |  |
| (**N**) | NRRL  2322 | - | - | 7 |  |  |  |
| *A. brasiliensis* (**N**) | CBS  626.66 | - | - | 1 |  |  |  |
| (**N**) | NRRL 3536 = ATCC 9642 | - | - | 2 |  | 1 | 2 |
| (**N**) | CBS  733.88 | - | - | 2 | 1 |  | 3 |
| *A. carbonarius* (**N**) | NRRL 67 | - | + | 1 |  |  |  |
|  | NRRL 368 | - | + | 1 |  |  |  |
|  | NRRL 369 | - | + |  |  | 1 |  |
| *A. niger*  **(A)**^c^ | CBS 102.12 | + | + |  |  | 1 | 1 |
|  | CBS 108.47 | + | - | 1 |  |  |  |
|  | CBS 127.48 | - | + |  |  | 1 |  |
|  | CBS 262.65 | + | - | 1 |  |  |  |
|  | CBS  630.78 | + | - |  |  | 1 |  |
|  | **CBS 513.88**^d^ | + | + |  | 1 | 1 |  |
|  | IFO 4122 | + | + | 1 |  |  |  |
|  | IFO 6082 | + | + | 1 |  |  |  |
|  | IFO 8876 | - | + | 1 |  |  |  |
|  | IFO  8877 | - | + | 1 |  |  |  |
|  | IMI 016141 | + | + | 1 |  |  |  |
|  | **NRRL 3** | + | - | 10 | 54 | 26 | 2 |
| **(A)** | NRRL 321 | - | - | 1 |  |  |  |
|  | NRRL 326 | + | - | 2 |  | 1 | 1 |
|  | **NRRL 328 = ATCC 1015** | + | - | 3 | 1 | 1 | 1 |
|  | NRRL 330 | + | - | 2 | 2 | 1 |  |
|  | NRRL 334 | + | - | 3 |  | 3 |  |
| **(A)** | NRRL 335 | - | - | 1 |  |  |  |
|  | NRRL 337 | + | + | 11 |  | 5 | 1 |
| **(A)** | NRRL 340 | - | - | 1 |  |  |  |
|  | **NRRL 350** | + | - | 5 | 1 | 1 | 1 |
|  | NRRL 341 | + | - |  |  | 3 |  |
|  | NRRL 363 | - | + | 1 |  |  |  |
|  | NRRL  364 | + | - | 1 |  | 1 |  |
|  | NRRL 372 | + | - | 2 |  |  |  |
|  | NRRL 566 | + | - | 1 | 1 | 1 | 1 |
| **(A)** | NRRL 567 | + | - | 10 |  |  |  |
| **(A)** | NRRL 593 | - | - | 1 |  |  |  |
| **(A)** | NRRL 595 | - | - | 2 |  |  | 1 |
|  | NRRL 599 | + | - | 31 |  |  | 6 |
| **(A)** | NRRL 604 | + | - | 1 |  |  |  |
|  | NRRL 611 | + | - | 1 |  |  |  |
|  | NRRL 615 | + | - | 1 |  |  |  |
| **(A)** | NRRL 2001 | + | - | 9 |  |  |  |
|  | **NRRL 2270** | + | - | 72 | 1 | 1 | 1 |
|  | NRRL 3112 | + | + |  | 10 | 4 |  |
|  | **NRRL 3122** | + | + |  | 1 | 11 | 1 |
| *A. tubingensis* (**N**) | ATCC 26036 | NT | NT | 1 |  |  |  |
| (**N**) | CBS 115.50 | - | - | 1 |  |  |  |
| (**N**) | CBS 130.52 | - | - | 1 |  |  |  |
| (**N**) | NRRL 322 | - | - | 1 |  |  | 1 |
| (**N**) | NRRL 2295 | - | - | 3 |  | 1 | 1 |
|  | NRRL 3135 | - | - |  |  | 3 |  |
| *A. vadensis*  (**N**) | CBS 113365 | - | - |  | 1 | 2 | 1 |

^a^ Strains with an (**N)** were originally identified as *Aspergillus niger*, but proved to be other black *Aspergillus* species.

^b^ The number under industrial applications is the times the strain has been used as the production organism and mentioned in scientific papers or listed in culture collection catalogues as an efficient producer of the industrial product.

^c^ Strains with an **(A)** are now referred to *A. awamori* as a phylogenetically distinct species.

^d^ Strains in bold have been full genome sequenced.
