# Supplementary material for: Fumonisin and Ochratoxin Production in Industrial Aspergillus niger Strains
Source: PLoS One. 2011 Aug 11;6(8):e23496. doi: 10.1371/journal.pone.0023496 (PMC3154942; doi:10.1371/journal.pone.0023496)
Supplement: Table S3 — contains strains of Aspergillus niger listed according to amounts of fumonisin B2, B4 and B6 produced. (DOC) [file pone.0023496.s003.doc]

**Table S3**. Supplementary material.

Fumonisin and ochratoxin production in industrial *Aspergillus niger* strains.

by

Jens C. Frisvad, Thomas O. Larsen, Ulf Thrane, Martin Meijer, Janos Varga, Robert A. Samson & Kristian Fog Nielsen

**Table S3**: Quantitative production of fumonisin B_2_, B_4_ and B_6_ (an isomer of fumonisin B_1_) by strains of *Aspergillus niger* on CYAS agar at 25°C after growth for 1 week (log_10_ to mass count).

| Strain | Log_10_ (Fumonisin B_2_) | Log_10_ (Fumonisin B_4_) | Log_10_ (Fumonisin B_6_) |
| --- | --- | --- | --- |
| NRRL 567*^a,b^ (A) | 6.40^c^ | 5.53 | 4.05 |
| CBS 113.33* | 6.09 | ND^d^ | ND |
| IBT 23191* | 6.09 | ND | ND |
| NRRL 357* | 6.00 | 4.78 | 3.55 |
| CBS 101706 | 5.99 | ND | ND |
| IBT 29276 (A) | 5.98 | 5.30 | 3.83 |
| IBT 29271 (A) | 5.94 | 5.33 | 3.78 |
| IBT 20959 (A) | 5.94 | +^e^ | + |
| IBT 29272 (A) | 5.93 | 5.31 | 3.80 |
| CBS 618.78 | 5.78 | 4.77 | ND |
| FRR 5181 | 5.76 | 4.67 | 3.43 |
| IFO 4067* | 5.75 | + | + |
| CBS 101705 | 5.74 | 4.50 | 3.44 |
| IBT 24634 | 5.70 | 5.88 | 4.40 |
| NRRL 1213* | 5.69 | 4.64 | ND |
| NRRL 2001* (A) | 5.65 | 4.67 | 3.27 |
| IBT 23366 | 5.61 | 4.52 | 3.34 |
| IBT 24631 | 5.60 | 4.65 | 3.18 |
| CBS 110.30* | 5.57 | 4.72 | 3.24 |
| IBT 23342 | 5.56 | ND | ND |
| NRRL 3122* | 5.55 | 4.64 | + (on YES agar) |
| NRRL 348* (A) | 5.52 | 4.81 | ND |
| NRRL 604* (A) | 5.52 | 4.37 | ND |
| IMI 016141 | 5.51 | 4.26 | 3.21 |
| NRRL 6408 (A) | 5.49 | 4.40 | ND |
| CBS 113.52* | 5.48 | 4.20 | 3.15 |
| CBS 126.48* | 5.47 | 4.67 | ND |
| IBT 23687 | 5.47 | 4.08 | 3.21 |
| NRRL 599* | 5.40 | 4.55 | 2.89 |
| NRRL 350* | 5.40 | 4.05 | ND |
| NRRL 611* | 5.39 | 4.55 | ND |
| NRRL 326* | 5.35 | + | + |
| NRRL 2270* | 5.33 | 4.80 | 3.98 |
| CBS 513.88* | 4.79 | 4.06 | ND |
| NRRL 330* | 5.30 | 4.00 | 2.94 |
| NRRL 511* | 5.30 | 4.03 | ND |
| NRRL 1278* | 5.30 | 3.96 | 2.58 |
| CBS 131.52* | 5.30 | 4.09 | ND |
| IBT 29331 | 5.30 | 4.46 | ND |
| CBS 101883 | 5.30 | 3.77 | 2.85 |
| CBS 113.50* | 5.30 | 3.23 | 2.40 |
| ITEM 7092 | 5.30 | 3.96 | ND |
| ITEM 7091 | 5.30 | 3.90 | ND |
| IBT 20369 | 5.29 | ND | ND |
| IBT 16764 | 5.26 | ND | ND |
| NRRL 615* | 5.24 | 4.90 | 4.11 |
| CBS 769.97 | 5.23 | 4.10 | 2.70 |
| IMI 015954* | 5.21 | 4.00 | 3.24 |
| NRRL 372* (A) | 5.21 | 4.26 | ND |
| IBT 24633 | 5.18 | 3.87 | 3.11 |
| IBT 27294 | 5.17 | 4.34 | ND |
| IBT 14491 | 5.17 | ND | ND |
| IBT 29226 | 5.13 | 4.31 | 2.99 |
| CBS 117.52* | 5.11 | ND | ND |
| IBT 3H7 | 5.10 | ND | ND |
| IBT 6E3 | 5.09 | ND | ND |
| IBT 29225 | 5.07 | 4.02 | 3.11 |
| NRRL 334 | 5.06 | ND | ND |
| NRRL 612* | 5.04 | 4.21 | ND |
| NRRL 3122* | 5.01 | + | + |
| IBT 12710 | 5.01 | + | + |
| NRRL 3112* | 5.01 | 4.21 | 3.08 |
| IBT 25744 | 5.00 | 3.76 | 2.81 |
| IBT 28104 | 5.00 | 3.97 | 2.78 |
| CBS 112.52* | 5.00 | 3.86 | ND |
| NRRL 364* | 4.97 | 3.19 | ND |
| IBT 12707 | 4.96 | ND | ND |
| IBT 29173 | 4.96 | 4.14 | 2.98 |
| IBT 23432 | 4.95 | 3.72 | 2.53 |
| IBT 23337 | 4.93 | ND | ND |
| IBT 29183 | 4.93 | 4.26 | 2.81 |
| IBT 25753 | 4.91 | 4.04 | ND |
| IBT 20306 | 4.91 | ND | ND |
| IBT 29185 | 4.90 | 4.00 | 2.87 |
| ITEM 4502 | 4.90 | 3.21 | ND |
| CBS 117785 (A) | 4.87 | 3.22 | ND |
| IBT 29336 | 4.87 | 3.33 | ND |
| IBT 3A2 | 4.86 | ND | ND |
| IBT 29187 | 4.84 | 4.23 | 2.72 |
| NRRL 566* | 4.83 | 4.69 | ND |
| IBT 3H3 | 4.83 | ND | ND |
| IBT 29181 | 4.82 | 4.16 | 2.66 |
| IBT 3H4 | 4.80 | ND | ND |
| IBT 29179 | 4.80 | 4.18 | 2.62 |
| IBT 1B7 | 4.79 | 3.74 | 2.52 |
| IBT 29195 | 4.79 | ND | 2.63 |
| IBT 29208 | 4.79 | 3.90 | 2.73 |
| IBT 29205 | 4.77 | 4.08 | 2.69 |
| NRRL 328* | 4.76 | + | + |
| IBT 29211 | 4.76 | 4.06 | 2.54 |
| IBT 29180 | 4.76 | 4.14 | 2.48 |
| IBT 29178 | 4.76 | 4.00 | 2.64 |
| NRRL 362* (A) | 4.74 | 3.90 | ND |
| IBT 29210 | 4.74 | 4.10 | 2.56 |
| CBS 623.78* | 4.72 | 3.71 | ND |
| IBT 29194 | 4.70 | 4.07 | 2.54 |
| IBT 29215 | 4.70 | 4.04 | 2.48 |
| IBT 29167 | 4.70 | 3.94 | 2.83 |
| IBT 29192 | 4.69 | 4.00 | 2.57 |
| IBT 29203 | 4.67 | 4.05 | 2.57 |
| IBT 29224 | 4.67 | 3.86 | 2.42 |
| NRRL 3* | 4.61 | + | + |
| IBT 1F1 | 4.59 | ND | ND |
| IBT 29197 | 4.58 | 3.74 | ND |
| IBT 1F7 | 4.56 | 3.57 | 1.93 |
| IBT 3E2 | 4.54 | ND | ND |
| IBT 1F6 | 4.48 | ND | ND |
| IBT 29337 | 4.48 | 3.50 | ND |
| IBT 21805 | 4.43 | 2.01 | 2.52 |
| CBS 101698 | 4.36 | ND | ND |
| IBT 20991 | 4.33 | 2.83 | 2.37 |
| CBS 113.30* (A) | 4.33 | 4.18 | ND |
| IBT 18599 | 4.31 | 2.58 | 2.27 |
| IBT 26774 | 4.31 | 3.47 | 2.13 |
| IBT 19558 | 4.31 | ND | ND |
| CBS 109.30* (A) | 4.30 | 4.31 | ND |
| CBS 121.28* | 4.24 | 4.00 | ND |
| IBT 1B6 | 4.23 | 2.46 | ND |
| IBT 4D4 | 4.23 | ND | ND |
| IBT 23680 | 4.23 | ND | ND |
| CBS 107.80* (A) | 4.22 | 4.18 | 1.90 |
| CBS 108.47* | 4.16 | 4.04 | ND |
| CBS 117.80* (A) | 4.15 | 4.04 | ND |
| CBS 563.65 | 4.15 | 3.77 | ND |
| CBS 108.80* | 4.15 | 4.10 | ND |
| CBS 124.48* | 4.05 | 2.99 | ND |
| IMI 041871* | 3.94 | 3.20 | ND |
| CBS 111.30* (A) | 3.93 | 3.98 | ND |
| ITEM 7097 (A) | 3.91 | 1.91 | 1.98 |
| CBS 112.30* | 3.86 | 3.85 | ND |
| IBT 5767 | 3.73 | 3.97 | ND |
| IBT 20182 | 3.59 | 1.88 | ND |
| CBS 555.65* (A) | 3.40 | 2.74 | ND |
| CBS 118.36* | 3.17 | 3.03 | ND |
| CBS 630.78* | 2.98 | 2.64 | ND |
| CBS 114.50* | 2.94 | 2.10 | ND |
| CBS 102.12* (A) | 2.90 | 2.46 | ND |
| IBT 29479 | 2.72 | 2.08 | ND |
| CBS 122.55 (A) | 2.71 | 1.48 | ND |
| CBS 263.65* | 2.69 | 1.46 | ND |
| IBT 29333 | 2.60 | 2.05 | ND |
| CBS 118725 (A) | 2.57 | ND | ND |
| IBT 25747 | 2.51 | 1.61 | ND |
| CBS 112.32* | 2.46 | 2.24 | ND |
| CBS 116.52* | 2.45 | ND | ND |
| IBT 29334 | 2.40 | 1.93 | ND |
| IBT 29335 | 2.40 | ND | ND |
| IBT 29329 | 2.36 | ND | ND |
| IBT 29342 | 2.34 | ND | ND |
| IBT 23744 | 2.23 | ND | ND |
| CBS 103.66 | 2.22 | 1.76 | ND |
| CBS 101708* | 2.22 | 1.77 | ND |
| IBT 29332 | 2.18 | 1.85 | ND |
| CBS 101700* | 2.14 | ND | ND |
| IBT 29343 | 2.10 | ND | ND |
| IBT 26773 | 2.07 | ND | ND |
| CBS 101703* | 2.05 | ND | ND |
| IBT 27302 | 2.03 | ND | ND |
| IBT 25752 | 1.56 | 1.94 | ND |

^a^:* indicates industrial strains (Further industrial strains tested positive for fumonisins are: ATCC 1015, ATCC 11414, N400, N402, AB 4.1).

^b^ Strains belonging to the *A. awamori* clade (phylogenetic species) are marked with an A in parenthesis.

^c^ The values are the maximal values detected. Several fumonisin measurements were repeated (biological replicates) and in those cases the standard deviation was from 5-25% of the average value. Several measurements were also done on YES agar with similar results, i.e. NRRL 567 was the best producer of fumonisins on both CYAS and YES agars.

^d^ ND: not detected

^e^ a + means that the mycotoxin was detected qualitatively, but not quantified
