# Supplementary material for: Fumonisin and Ochratoxin Production in Industrial Aspergillus niger Strains
Source: PLoS One. 2011 Aug 11;6(8):e23496. doi: 10.1371/journal.pone.0023496 (PMC3154942; doi:10.1371/journal.pone.0023496)
Supplement: Table S4 — is a referenced list of citric acid producers, transformation hosts, enzyme, single cell protein, and animal feed producer strains, strains used for biotransforming natural products and strains producing lipids in Aspergillus section Nigri. This table is followed by 299 supplementary references. (DOC) [file pone.0023496.s004.doc]

**Table S4**. Supplementary material.

Fumonisin and ochratoxin production in industrial *Aspergillus niger* strains.

by

Jens C. Frisvad, Thomas O. Larsen, Ulf Thrane, Martin Meijer, Janos Varga, Robert A. Samson & Kristian Fog Nielsen

**Table S4**: Industrial uses of *Aspergillus* section *Nigri* strains:

**S4a**. Citric acid production by known culture collection strains. Fumonisin produced by all strains of *A. niger*, except where otherwise noted.

| Species | Culture collection accession number | Examples of waste substrates used and notes | References |
| --- | --- | --- | --- |
| *A. acidus* | NRRL 2322 = NRRL 2354 | Beet molasses, figs and fig water, wet corn distillers grains | 1-7 |
| *A. acidus* | CBS 126.52 | Koji | 8 |
| *A. brasiliensis* | CBS 626.66 |  | 9 |
| *A. brasiliensis* | CBS 733.88 |  | 9-11 |
| *A. brasiliensis* | NRRL 3536 | Whey | 9,10,12 |
| *A. carbonarius* | NRRL 67 | Beet molasses | 13 |
| *A. carbonarius* | NRRL 368 |  | 7 |
| *A. niger* | CBS 108.47 | Ascorbic acid, weak citric acid | 9 |
| *A. niger* | CBS 101700 = IFO 6082 |  | 8 |
| *A. niger* | CBS 101701 = IFO 8877 |  | 8 |
| *A. niger* | CBS 101704 = IFO 8876 |  | 8 |
| *A. niger* | CBS 101708 = IFO 4122 |  | 8 |
| *A. niger* | IMI 016141 | Fumaric acid, weak citric acid | 9 |
| *A. niger* | NRRL 3 | Citric acid, gluconic acid, oxalic acid | 4,9,14-21 |
| *A. niger* | NRRL 321 | Fumonisin not found | 22-23 |
| *A. niger* | NRRL 326 |  | 9,10,22,23 |
| *A. niger* | NRRL 328 (= ATCC 1015 = NRRL 350 = NRRL 511 = NRRL 1278) | Apple and grape pomace, pineapple waste | 7, 24-27 |
| *A. niger* | NRRL 330 | Soy-residue | 28-29 |
| *A. niger* | NRRL 334 |  | 30,31, US Pat. 4,040,906 |
| *A. niger* | NRRL 335 | Fumonisin not found | 22-23 |
| *A. niger* | NRRL 337 | Beet waste, brewery spent grain, pineapple waste, potato peels | 1,9,10,32-42 |
| *A. niger* | NRRL 340 | Fumonisin not found | 22-23 |
| *A. niger* | NRRL 350 (= ATCC 1015) (see also derived strain NRRL 2270) |  | 7,22-23,26,43-44 |
| *A. niger* | NRRL 363 = IFO 4034 |  | 8 |
| *A. niger* | NRRL 364 | Orange waste, citric acid and oxalic acid | 45 |
| *A. niger* | NRRL 372 |  | 7,22-23 |
| *A. niger* | NRRL 567 | Apple and grape pomace, kiwifruit peel,  orange processing waste, peat moss | 1,4,7,22-23,25-26,45-49 |
| *A. niger* | NRRL 593 | Fumonisin not found | 22-23 |
| *A. niger* | NRRL 595 | Date syrup, fumonisin not found, heavy metal treatment | 50-52 |
| *A. niger* | NRRL 599, and  ATCC 64065, mutant of NRRL 599 | Beet molasses, brewery waste, cane molasses, carob pod, cotton waste, date syrup, molasses, orange processing waste, whey,  wood hemicelluloses | 1,4,7,25-26,31,42,51-75 |
| *A. niger* | NRRL 604 |  | 22 |
| *A. niger* | NRRL 611 |  | 23 |
| *A. niger* | NRRL 615 |  | 23 |
| *A. niger* | NRRL 2001 | Apple and grape pomace, cassava bagasse | 7,22,25-26,42,76-80 |
| *A. niger* | NRRL 2270 | Apple and grape pomace | 7,25-26, 81-147 |
| *A. niger* | CBS 262.65 | Mussel processing effluent | 148 |
| *A. tubingensis* | ATCC 26036 |  | 149 |
| *A. tubingensis* | CBS 115.50 |  | 9,10,150 |
| *A. tubingensis* | CBS 130.52 |  | 9 |
| *A. tubingensis* | NRRL 322 |  | 151 |
| *A. tubingensis* | NRRL 2295 |  | 152-154 |

**S4b**. *Aspergillus* section *Nigri* culture collection strains used as transformation hosts for protein production. All *A. niger* strains listed , except NRRL 595, produced fumonisins in pure culture.

| Species | Culture collection accession number | Notes | References |
| --- | --- | --- | --- |
| *A. acidus* | CBS 115.52 = ATCC 11358 (and mutants and transformants derived from it) |  | 155-163 |
| *A. niger* | NRRL 3 (and mutants and transformants derived from it) | Direct enzyme production (ref. 212) | 162-212 |
| *A. niger* | NRRL 328 (=ATCC 1015) |  | 213 |
| *A. niger* | NRRL 330 (and mutants and transformants derived from it) |  | 214-215 |
| *A. niger* | NRRL 3112 (and mutants and transformants derived from it |  | 215-223 |
| *A. vadensis* | CBS 113365 |  | 224 |

**S4c**. Production of enzymes, single cell protein or animal feed by strains of *Aspergillus niger* and closely related species (only a restricted list). All *A. niger* strains listed produced fumonisin in pure culture.

| Species | Culture collection accession number | References |
| --- | --- | --- |
| *A. carbonarius* | NRRL 369 | US Patent 5667990 |
| *A. niger* | CBS 127.48 = NRRL 4869 | 225 |
| *A. niger* | CBS 630.78 | 226 |
| *A. niger* | NRRL 3 | 170,227-250 |
| *A. niger* | NRRL 334 | 251-253 |
| *A. niger* | NRRL 337 | 254-258 |
| *A. niger* | NRRL 341 | 259-261 |
| *A. niger* | NRRL 566 | 262 |
| *A. niger* | NRRL 3112 | 257,263-265 |
| *A. niger* | NRRL 3122 | 248,265-272 |
| *A. tubingensis* | NRRL 3135 | 273-275 |
| *A. vadensis* | IMI 142717 = CBS 113365 | 224,276 |

**S4e**. Biotransformations of natural products by strains of *Aspergillus niger* and related species. All strains of *A. niger* listed produced fumonisin.

| Species | Culture collection accession number | references |
| --- | --- | --- |
| *A. brasiliensis* | CBS 733.88 | 277-279 |
| *A. brasiliensis* | NRRL 3536 | 280-281 |
| *A. niger* | CBS 102.12 | 282 |
| *A. niger* | CBS 563.65 = NRRL 341 | 283 |
| *A. niger* | NRRL 3 | 284-285 |
| *A. niger* | NRRL 326 | 286 |
| *A. niger* | NRRL 328 | 287-288 |
| *A. niger* | NRRL 337 | 289 |
| *A. niger* | NRRL 595 | 290 |
| *A. niger* | NRRL 599 | 291-296 |
| *A. niger* | NRRL 3122 | 297 |
| *A. tubingensis* | NRRL 322 | 298 |
| *A. vadensis* | CBS 113365 | 224 |

**S4f**. Lipid production by strains of *Aspergillus* section *Nigri*. Both strains produced fumonisin in pure culture.

| Species | Culture collection accession number | Reference |
| --- | --- | --- |
| *A. niger* | NRRL 363 | 299 |
| *A. niger* | NRRL 364 | 299 |

Supplementary Reference List to Table S4

1. Tran,C.T., Sly,L.I., & Mitchell,D.A. Selection of a strain of *Aspergillus* for the production of citric acid from pineapple waste in solid-state fermentation. *World J. Microbiol. Biotechnol.* **14**, 399-404 (1998).

2. Roukas,T. Citric and gluconic acid production from fig by *Aspergillus niger* using solid-state fermentation. *J. Ind. Microbiol. Biotechnol.* **25**, 298-304 (2000).

3. Roukas,T. & Liakopoulou-Kyriakides, M. Optimization study for the production of citric and gluconic acid from fig water extract by *Aspergillus niger* in surface fermentation. *Food Biotechnol.* **16**, 17-28 (2002).

4. Xie,G. & West,T.P. Citric acid production by *Aspergillus niger* on wet corn distillers grains. *Lett. Appl. Microbiol.* **43**, 269-273 (2006).

5. Bruchman, E.E. Zur Wirkung des Wasserstoffperoxids auf die Citronensaureanhäufung durch *Aspergillus niger*. *Naturwissenschaften* **53**, 226-230 (1966).

6. Clement, M.T. Citric acid fermentation of beet molasses by *Aspergillus niger* in submerged culture. *Can. J. Technol.* **30**, 82-88 (1952).

7. Maddox, I.S., Hossain, M., & Brooks, J.D. The effect of methanol on citric acid production from galactose by *Aspergillus niger*. *Appl. Microbiol. Biotechnol.* **23**, 203-205 (1986).

8. Oyashiki, H. *et al.* Use of Koji Prepared with a high citric-acid producing mutant of *Aspergillus usamii* as a raw-material for sake brewing. *J. Ferment. Tech.* **66**, 111-115 (1988).

9. Pintado, J., Murado, M.A., Gonzalez, P., Miron, J., & Pastrana, L. Joint effect of nitrogen and phosphorus concentrations on citric-acid production by different strains of *Aspergillus niger* grown on an effluent. *Biotechnol. Lett.* **15**, 1157-1162 (1993).

10. Pintado, J., Torrado, A., Gonzalez, M.P., & Murado, M.A. Optimization of nutrient concentration for citric acid production by solid-state culture of *Aspergillus niger* on polyurethane foams. *Enzyme Microb. Tech.* **23**, 149-156 (1998).

11. Pallares, J., Rodriguez, S., & Sanroman, A. Citric acid production in submerged and solid state culture of *Aspergillus niger*. *Bioproc. Eng.* **15**, 31-33 (1996).

12. El-Holi, M.A. & Al-Delaimy, K.S. Citric acid production from whey with sugars and additives by *Aspergillus niger*. *Afr. J. Biotechnol.* **2**, 356-359 (2003).

13. Gerhardt, P., Dorrell, W.W., & Baldwin,I .L. Citric acid fermentation of beet molasses. *J. Bacteriol.* **52**, 555-564 (1946).

14. Blom, R.H. *et al.* Sodium gluconate production - fermentation with *Aspergillus niger*. *Ind. Eng. Chem.* **44**, 435-440 (1952).

15. Somkuti, G.A. & Bencivengo, M.M. Citric acid fermentation in whey permeate. *Dev. Ind. Microbiol.* **22**, 557-563 (1981).

16. Bercovitz, A., Peleg, Y., Battat, E., Rokem, J.S., & Goldberg, I. Localization of pyruvate-carboxylase in organic acid-producing *Aspergillus* strains. *Appl. Environ. Microbiol.* **56**, 1594-1597 (1990).

17. Dronawat, S.N., Svihla, C.K., & Hanley, T.R. The effects of agitation and aeration on the production of gluconic acid by *Aspergillus niger*. *Appl. Biochem. Biotechnol.* **51-2**, 347-354 (1995).

18. Meijer, S., Panagiotou, G., Olsson, L., & Nielsen, J. Physiological characterization of xylose metabolism in *Aspergillus niger* under oxygen-limited conditions. *Biotechnol. Bioeng.* **98**, 462-475 (2007).

19. de Jongh, W.A. & Nielsen,J . Enhanced citrate production through gene insertion in *Aspergillus niger*. *Metab. Eng.* **10**, 87-96 (2008).

20. Solar, T., Tursic, J., & Legisa, M. The role of glucosamine-6-phosphate deaminase at the early stages of *Aspergillus niger* growth in a high-citric-acid-yielding medium. *Appl. Microbiol. Biotechnol.***78**, 613-619 (2008).

21. Strasser, H., Burgstaller, W., & Schinner, F. High-yield production of oxalic-acid for metal leaching processes by *Aspergillus niger*. *FEMS Microbiol. Lett.***119**, 365-370 (1994).

22. Moyer, A.J. Effect of Alcohols on the mycological production of citric acid in surface and submerged culture .1. Nature of the alcohol effect. *Appl. Microbiol* **1**, 1-7 (1953).

23. Moyer, A.J. Effect of alcohols on the mycological production of citric acid in surface and submerged culture .2. Fermentation of crude carbohydrates. *Appl. Microbiol.* **1**, 7-13 (1953).

24. Hang, Y.D. & Woodams, E.E. Effect of substrate moisture-content on fungal production of citric acid in a solid-state fermentation system. *Biotechnol. Lett.* **9**, 183-186 (1987).

25. Hang, Y.D. & Woodams, E.E. Grape pomace - a novel substrate for microbial-production of citric acid. *Biotechnol. Lett.* **7**, 253-254 (1985).

26. Hang, Y.D. & Woodams, E.E. Apple pomace - a potential substrate for citric-acid production by *Aspergillus niger*. *Biotechnol. Lett.* **6**, 763-764 (1984).

27. Lima, V.L.A.G., Stamford, T.L.M., & Salguiero, A.-A. Produção de ácido cítrico utilizando *Aspergillus niger* em resíduo de abacaxi (*Ananas comosus* (L) Merril) pro fermentação em estudo sólido. *Arq. Biol. Tecnol.* **38**, 773-783 (1995).

28. Kurbanoglu, E.B. & Kurbanoglu, N.I. Production of citric acid from ram horn hydrolysate by *Aspergillus niger*. *Process Biochem.* **38**, 1421-1424 (2003).

29. Khare,S.K., Jha,K., & Gandhi,A.P. Citric acid production from okara (soy-residue) by solid-state fermentation. *Biores. Technol.* **54**, 323-325 (1995).

30. Gunde-Cimerman, N., Cimerman, A., & Perdih, A. *Aspergillus niger* mutants for bioconversion of apple distillery wastes. *Enzyme Microb. Tech.* **8**, 166-170 (1986).

31. Mehyar, G.F., Delaimy, K.S., & Ibrahim, S.A. Citric acid production by *Aspergillus niger* using date-based medium fortified with whey and additives. *Food Biotechnol.* **19**, 137-144 (2005).

32. Lewis, K.F. & Weinhouse, S. Studies on the mechanism of citric acid production in *Aspergillus niger*. *J. Am. Chem. Soc.***73**, 2500-2503 (1951).

33. Elmayerg, H. & Scharer, J.M. Physiological studies on *Aspergillus niger* fermentation with polymer additive. *J. Gen. Appl. Microbiol.***19**, 385-392 (1973).

34. Hang, Y.D. Fungal treatment of beet waste. *Prog. Wat. Technol.* **8**, 325-327 (1976).

35. Hang, Y.D. & Woodams, E.E. Characterization of baked bean processing wastewater and its assimilation by *Aspergillus foetidus*. *J. Food Sci.* **44**, 1548-1549 (1979).

36. Hang, Y.D., Splittstoesser, D.F. & Woodams, E.E. Utilization of brewery spent grain liquor by *Aspergillus niger*. *Appl. Microbiol.* **30**, 879-880 (1975).

37. Hang, Y.D., Splittstoesser, D.F., Woodams, E.E., & Sherman, R.M. Citric acid fermentation of brewery waste. *J. Food Sci.* **42**, 383-384 (1977).

38. Chen, H.C. Optimizing the concentrations of carbon, nitrogen and phosphorus in a citric acid fermentation with response surface method. *Food Biotechnol.* **10**, 13-27 (1996).

39. Chen, H.C. Citric acid production by *Aspergillus foetidus* in batch and fed-batch cultures. *Food Biotechnol.* **7**, 221-234 (1993).

40. Chen, H.C. Response-surface methodology for optimizing citric acid fermentation by *Aspergillus foetidus*. *Process Biochem.* **29**, 399-405 (1994).

41. Tran, C.T. & Mitchell, D.A. Pineapple waste - a novel substrate for citric acid production by solid-state fermentation. *Biotechnol. Lett.* **17**, 1107-1110 (1995).

42. Ousmanova, D. & Parker, W. Fungal generation of organic acids for removal of lead from contaminated soil. *Water Air and Soil Pollution* **179**, 365-380 (2007).

43. Currie, J.N. The citric acid fermentation of *Aspergillus niger*. *J. Biol. Chem.* **31**, 15-37 (1917).

44. Delima, V.L.A.G., Stamford, T.L.M. & Salgueiro, A.A. Citric acid production from pineapple waste by solid state fermentation using *Aspergillus niger*. *Arq. Biol Tecnol.* **38**, 773-783 (1995).

45. Aravantinoszafiris, G., Tzia, C., Oreopoulou, V., & Thomopoulos, C.D. Fermentation of orange processing wastes for citric acid production. *J. Sci. Food Agric.* **65**, 117-120 (1994).

46. Hang, Y.D. Microbial-Production of citric acid in fixed-bed column bioreactors. *Biotechnol. Lett.* **10**, 421-426 (1988).

47. Hang, Y.D., Luh, B.S., & Woodams, E.E. Microbial production of citric acid by solid-state fermentation of kiwifruit peel. *J. Food Sci.* **52**, 226-227 (1987).

48. Kim, J.W., Barrington, S., Sheppard, J., & Lee, B. Nutrient optimization for the production of citric acid by *Aspergillus niger* NRRL 567 grown on peat moss enriched with glucose. *Proc. Biochem.* **41**, 1253-1260 (2006).

49. Barrington, S. & Kim, J.W. Response surface optimization of medium components for citric acid production by *Aspergillus niger* NRRL 567 grown in peat moss. *Biores. Technol.* **99**, 368-377 (2008).

50. Saad, M.M. Chelating ability of the chitosan-glucan complex from *Aspergillus niger* NRRL 595 biomass recycling in citric acid production. *Res. J. Agric. Biol. Sci* **2**, 132-136 (2006).

51. Saad, M.M. Citric acid production from pretreating crude date syrup by *Aspergillus niger* NRRL 595. .*J Appl. Sci. Res.* **2**, 74-79 (2006).

52. El-Abyad, M.S., Hamissa, F.A., & Gadd, A.S. Growth kinetics of heavy metal adapted *Aspergillus niger* NRRL 595. *Afr. J. Mycol. Biotechnol.* **4**, 59-69 (1996).

53. Hamissa,F.A. & Samie,M.E.D.A. Effect of acetic and trans-aconitic acids on citric acid product by *Aspergillus niger*. *Acta Microbiol. Polon.* **25**, 227-231 (1976).

54. Shadafza, D., Ogawa, T., & Fazeli, A. Comparison of citric-acid production from beet molasses and date syrup with *Aspergillus niger.* *J. Ferment. Tech.* **54**, 67-75 (1976).

55. Hamissa, F.A. & Radwan, A. Production of citric acid from cane molasses on a semi-pilot scale. *J. Gen. Appl. Microbiol.* **23**, 325-329 (1977).

56. Hamissa, F.A., Mabrouk ,S.S., & Abdelfattah, A.F. Formation of polygalacturonase activities in citric acid fermentation by *Aspergillus niger*-599. *J. Gen. Appl.Microbiol.* **23**, 23-27 (1977).

57. Kiel, H., Guvrin, R., & Henis, Y. Citric acid fermentation by *Aspergillus niger* on low sugar concentrations and cotton waste. *Appl. Environ. Microbiol.* **42**, 1-4 (1981).

58. Heinrich, M. & Rehm, H.J. Formation of gluconic acid at low pH-values by free and immobilized *Aspergillus niger* cells during citric acid fermentation. *Eur. J. Appl. Microbiol. Biotechnol.* **15**, 88-92 (1982).

59. Hossain, M., Brooks, J.D., & Maddox, I.S. Production of citric acid from whey permeate by fermentation using *Aspergillus niger*. *New Zealand J. . Dairy Sci. and Technol.* **18**, 161-168 (1983).

60. Hossain, M., Brooks, J.D., & Maddox, I .S. The Effect of the sugar source on citric acid production by *Aspergillus niger*. *Appl. Microbiol. Biotechnol.***19**, 393-397 (1984).

61. Vaija, J. & Linko, P. Continuous citric acid production by immobilized *Aspergillus niger* - reactor performance and fermentation kinetics. *J. Mol.Catal.* **38**, 237-253 (1986).

62. Roukas, T. & Harvey, L. The Effect of pH on production of citric and gluconic acid from beet molasses using continuous culture. *Biotechnol. Lett.* **10**, 289-294 (1988).

63. Roukas, T. & Alichanidis, E. Citric acid production from beet molasses by cell recycle of *Aspergillus niger*. *J. Ind. Microb.* **7**, 71-73 (1991).

64. Khare, S.K., Jha,K., & Gandhi, A.P. Use of agarose-entrapped *Aspergillus niger* cells for the production of citric acid from soy whey. *Appl.Microbiol. Biotechnol.***41**, 571-573 (1994).

65. Khosravi, D.K., Zoghi, A., Alavi, S.A., & Fatemi, S.S.A. Application of Plackett Burman design for citric acid production from pretreated and untreated wheat straw. *Iran. J. Chem. Chem. Eng.-Int. Eng. Ed.***27**, 91-104 (2008).

66. Rivas, B., Toizrado, A., Torre, P., Converti, A., & Dominguez, J.M. Submerged citric acid fermentation on orange peel autohydrolysate. *J.Agric. Food Chem.* **56**, 2380-2387 (2008).

67. Xie, G. & West, T.P. Citric acid production by *Aspergillus niger* ATCC 9142 from a treated ethanol fermentation co-product using solid-state fermentation. *Lett. Appl. Microbiol.* **48**, 639-644 (2009).

68. Doelger, W.P. & Prescott, S.C. Citric acid fermentation. *Ind. Eng. Chem.***26**, 1142-1149 (1934).

69. Ogawa, T. & Fazeli, A. Additive Effect of ferrocyanide treatment and step change of pH on citric acid production from Iranian beet molasses with *Aspergillus niger*. *J. Ferment. Tech.* **54**, 63-66 (1976).

70. Roukas,T. Production of citric acid from beet molasses by immobilized cells of *Aspergillus niger*. *J. Food Sci.* **56**, 878-880 (1991).

71. Maddox, I.S., Spencer, K., Greenwood, J.M., Dawson, M.W., & Brooks, J.D. Production of citric ccid from sugars present in wood hemicellulose using *Aspergillus niger* and *Saccharomycopsis lipolytica*. *Biotechnol. Lett.* **7**, 815-818 (1985).

72. Roukas, T. & Kotzekidou, P. Production of citric acid from brewery wastes by surface fermentation using *Aspergillus niger*. *J. Food Sci.* **51**, 225-228 (1986).

73. Dawson, M.W., Maddox, I.S., & Brooks, J.D. Evidence for nitrogen catabolite repression during citric acid production by *Aspergillus niger* under phosphate-limited growth-conditions. *Biotechnol. Bioeng.* **33**, 1500-1504 (1989).

74. Roukas, T. Carob pod: A new substrate for citric acid production by *Aspergillus niger*. *Appl. Biochem. Biotechnol.***74**, 43-53 (1998).

75. Roukas, T. Citric acid production from carob pod extract by cell recycle of *Aspergillus niger* ATCC 9142. *Food Biotechnol.***12**, 91-104 (1998).

76. Karow, E.O. & Waksman, S.A. Production of citric acid in submerged culture. *Ind. Eng. Chem.* **39**, 821-825 (1947).

77. Hang, Y.D. & Woodams, E.E. Enzymatic enhancement of citric acid production by *Aspergillus niger* from corn cobs. *Lebensm.-Wiss. Technol.* **34**, 484-486 (2001).

78. Hang,Y.D. & Woodams, E.E. Corn husks: A potential substrate for production of citric acid by *Aspergillus niger*. *Lebensm.-Wiss. Technol.* **33**, 520-521 (2000).

79. Guilherme, A.A., Pinto, G.A.S., & Rodrigues, S. Optimization of trace metals Concentration on citric acid production by *Aspergillus niger* NRRL 2001. *Food Bioproc. Technol.* **1**, 246-253 (2008).

80. Vandenberghe, L.P.S., Soccol, C.R., Pandey, A., & Lebeault, J.M. Solid-state fermentation for the synthesis of citric acid by *Aspergillus niger*. *Biores.Technol.***74**, 175-178 (2000).

81. Shu, P. & Johnson, M.J. Effect of the Composition of the sporulation medium on citric acid production by *Aspergillus niger* in submerged culture. *J. Bacteriol.* **54**, 161-167 (1947).

82. Shu, P. & Johnson, M.J. The Interdependence of medium constituents in citric acid production by submerged fermentation. *J. Bacteriol.* **56**, 577-585 (1948).

83. Tomlinson, N., Campbell, J.J.R., & Trussell, P.C. The influence of zinc, iron, copper, and manganese on the production of citric acid by *Aspergillus niger*. *J. Bacteriol.* **59**, 217-227 (1950).

84. Bomstein, R.A. & Johnson, M.J. The mechanism of formation of citrate and oxalate by *Aspergillus niger*. *J. Biol. Chem.* **198**, 143-153 (1952).

85. Kitos, P.A., Campbell, J.J.R., & Tomlinson, N. Influence of temperature on the trace element requirements for citric acid production by *Aspergillus niger*. *Appl. Microbiol.* **1**, 156-159 (1953).

86. Shu, P., Funk, A., & Neish, A.C. Mechanism of citric acid formation from glucose by *Aspergillus niger*. *Can. J. Biochem. Physiol.* **32**, 68-80 (1954).

87. Clark, D.S. Submerged citric acid fermentation of ferrocyanide-treated cane molasses. *Biotechnol. Bioeng.* **4**, 17-21 (1962).

88. Clark, D.S. & Lentz, C.P. Submerged citric acid fermentation of beet molasses in tank-type fermenters. *Biotechnol. Bioeng.* **5**, 193-199 (1963).

89. Clark, D.S., Ito, K., & Tymchuk, P. Effect of potassium ferrocyanide on chemical composition of molasses mash used in citric acid fermentation. *Biotechnol. Bioeng*  **7**, 269-278 (1965).

90. Clark, D.S., Ito,K., & Horitsu,H. Effect of manganese and other heavy metals on submerged citric acid fermentation of molasses. *Biotechnol.Bioeng.* **8**, 465-471 (1966).

91. Millis, N.F., Palmer, B.M., & Trumpy, B.H. Effect of lipids on citric acid production by an *Aspergillus niger* mutant. *J. Gen. Microbiol.* **30**, 365-368 (1963).

92. Trumpy, B.H. & Millis, N.F. Nutritional requirements of an *Aspergillus niger* mutant for citric acid production. *J. Gen. Microbiol.* **30**, 381-393 (1963).

93. Ahmed, S.A., Anderson, J.G., & Smith, J.E. Mitochondrial activity during citric acid production by *Aspergillus niger*. *Trans. Brit. Mycol. Soc.* **59**, 51-61 (1972).

94. Wold, W.S.M. & Suzuki, I. Cyclic-AMP and citric acid accumulation by *Aspergillus niger*. *Biochem. Biophys. Res. Com.* **50**, 237-244 (1973).

95. Macris, B.J. Citric acid from purified carob sugars. *Biotechnol. Bioeng.***17**, 1373-1374 (1975).

96. Kubicek, C.P. & Rohr,M. Influence of manganese on enzyme synthesis and citric acid accumulation in *Aspergillus niger*. *Eur. J. Appl. Microbiol.* **4**, 167-175 (1977).

97. Kubicek, C.P. & Rohr, M. Role of Tricarboxylic-acid cycle in citric acid accumulation by *Aspergillus niger*. *Eur. J. Appl.Microbiol. Biotechnol.* **5**, 263-271 (1978).

98. Kubicek, C.P., Zehentgruber, O., & Rohr, M. Indirect method for studying the fine control of citric acid formation by *Aspergillus niger*. *Biotechnol. Lett.* **1**, 47-52 (1979).

99. Kubicek, C.P., Hampel, W., & Rohr, M. Manganese deficiency leads to elevated amino-acid pools in citric acid accumulating *Aspergillus niger*. *Arch. Microbiol.* **123**, 73-79 (1979).

100. Rohr, M., Stadler,P.J., Salzbrunn, W.O.J., & Kubicek, C.P. Improved method for characterization of citrate production by conidia of *Aspergillus niger*. *Biotechnol. Lett.***1**, 281-286 (1979).

101. Kubicek, C.P., Zehentgruber, O., Elkalak, H., & Rohr, M. Regulation of citric acid production by oxygen - effect of dissolved oxygen tension on adenylate levels and respiration in *Aspergillus niger*. *Eur. J. Appl. Microbiol. Biotechnol.* **9**, 101-115 (1980).

102. Kubicek, C.P. & Rohr, M. Regulation of citrate synthase from the citric acid-accumulating fungus, *Aspergillus niger*. *Biochim. Biophys. Acta* **615**, 449-457 (1980).

103. Roehr, M., Zehentgruber, O., & Kubicek, C.P. Kinetics of biomass formation and citric acid production by *Aspergillus niger* on pilot-plant scale. *Biotechnol. Bioeng.* **23**, 2433-2445 (1981).

104. Kubicek, C.P. & Rohr, M. Aconitase and citric acid fermentation by *Aspergillus niger*. *Appl. Environ. Microbiol.* **50**, 1336-1338 (1985).

105. Roehr, M., Kubicek, C.P., Zehentgruber, O., & Orthofer, R. Accumulation and partial reconsumption of polyols during citric acid fermentation by *Aspergillus niger*. *Appl.Microbiol. Biotechnol.***27**, 235-239 (1987).

106. Wolschek, M.F. & Kubicek, C.P. The filamentous fungus *Aspergillus niger* contains two ''differentially regulated'' trehalose-6-phosphate synthase-encoding genes, tpsA and tpsB. *J. Biol. Chem*. **272**, 2729-2735 (1997).

107. Kubicek, C.P. The role of sugar uptake and channelling for citric acid accumulation by *Aspergillus niger*. *Food Technol. Biotechnol.***36**, 173-175 (1998).

108. Habison, A., Kubicek, C.P., & Rohr, M. Phosphofructokinase as a regulatory enzyme in citric acid producing *Aspergillus niger*. *FEMS Microbiol. Lett.* **5**, 39-42 (1979).

109. Orthofer, R., Kubicek, C.P., & Rohr, M. Lipid-levels and manganese deficiency in citric acid producing strains of *Aspergillus niger*. *FEMS Microbiol.Lett.* **5**, 403-406 (1979).

110. Alobaidi, Z.S. & Berry, D.R. Camp concentration, morphological-differentiation and citric acid production in *Aspergillus niger*. *Biotechnol. Lett.* **2**, 5-10 (1980).

111. Alobaidi, Z.S. & Berry, D.R. Extended production of citric acid using an exchange filtration technique. *Biotechnol. Lett.* **1**, 221-224 (1979).

112. Anderson, J.G., Blain, J.A., Divers, M., & Todd, J.R. Use of the disk fermenter to examine production of citric acid by *Aspergillus niger*. *Biotechnol. Lett.* **2**, 99-104 (1980).

113. Kisser, M., Kubicek, C.P., & Rohr, M. Influence of manganese on morphology and cell-wall composition of *Aspergillus niger* during citric acid fermentation. *Arch. Microbiol.* **128**, 26-33 (1980).

114. Legisa, M., Cimerman, A., & Sterle, M. Germination of *Aspergillus niger* in a high citric acid yielding medium. *FEMS Microbiol. Lett.* **11**, 149-152 (1981).

115. Jernejc, K., Cimerman, A., & Perdih, A. Citric acid production in chemically defined media by *Aspergillus niger*. *Eur. J.Applied Microbiol. Biotechnol.***14**, 29-33 (1982).

116. Meixner, O., Mischak, H., Kubicek, C.P., & Rohr, M. Effect of manganese deficiency on plasma-membrane lipid-composition and glucose-uptake in *Aspergillus niger*. *FEMS Microbiol. Lett.* **26**, 271-274 (1985).

117. Mischak, H., Kubicek, C.P., & Rohr, M. Formation and location of glucose-oxidase in citric acid producing mycelia of *Aspergillus niger*. *Appl. Microbiol. Biotechnol.***21**, 27-31 (1985).

118. Legisa, M. & Mattey, M. Glycerol as an initiator of citric acid accumulation in *Aspergillus niger*. *Enzyme Microb. Tech.* **8**, 258-259 (1986).

119. Schreferl, G., Kubicek, C.P., & Rohr, M. Inhibition of citric acid accumulation by manganese ions in *Aspergillus niger* mutants with reduced citrate control of phosphofructokinase. *J. Bacteriol.* **165**, 1019-1022 (1986).

120. Legisa, M. & Mattey, M. Citrate Regulation of the change in carbohydrate degradation during the initial phase of the citric acid production by *Aspergillus niger.* *Enzyme Microb. Tech.* **10**, 33-36 (1988).

121. Legisa, M. & Kidric, J. Initiation of citric acid accumulation in the early stages of *Aspergillus niger* growth. *Appl. Microbiol. Biotechnol.* **31**, 453-457 (1989).

122. Xu, D.B., Madrid, C.P., Rohr, M., & Kubicek, C.P. The influence of type and concentration of the carbon source on production of citric acid by *Aspergillus niger*. *Appl. Microbiol. Biotechnol.* **30**, 553-558 (1989).

123. Jernejc, K., Cimerman, A., Vendramin, M., & Perdih, A. Lipids of a citric acid-producing *Aspergillus niger* strain grown in copper-supplemented and in manganese-supplemented media. *Appl. Microbiol. Biotechnol.* **32**, 699-703 (1990).

124. Berovic, M., Cimerman, A., Steiner, W., & Koloini, T. Submerged citric acid fermentation - rheological properties of *Aspergillus niger* broth in a stirred tank reactor. *Appl. Microbiol.Biotechnol.* **34**, 579-581 (1991).

125. Jaklitsch, W.M., Kubicek, C.P., & Scrutton, M.C. Intracellular location of enzymes involved in citrate production by *Aspergillus niger*. *Can. J. Microbiol.* **37**, 823-827 (1991).

126. Jernejc,K., Cimerman,A., & Perdih,A. Composition of *Aspergillus niger* mycelium during growth on productive and unproductive substrates. *Journal of Biotechnology* **25**, 341-348 (1992).

127. Sassi, G., Ruggeri, B., Specchia, V., & Gianetto, A. Citric acid production by *Aspergillus niger* with banana extract. *Biores. Technol.* **37**, 259-269 (1991).

128. Legisa, M. & Kidric, J. The influence of intracellular pH of spores on citric acid production by *Aspergillus niger*. *J. Biotechnol.* **20**, 313-318 (1991).

129. Steinbock, F.A. *et al.* Regulatory aspects of carbohydrate-metabolism in relation to citric acid accumulation by *Aspergillus niger*. *Acta Biotechnol.* **11**, 571-581 (1991).

130. Harmsen, H.J.M., Kubicekpranz, E.M., Rohr, M., Visser, J., & Kubicek, C.P. Regulation of 6-phosphofructo-2-kinase from the citric acid-accumulating fungus *Aspergillus niger*. *Appl. Microbiol. Biotechnol.* **37**, 784-788 (1992).

131. Yigitoglu, M. & Mcneil, B. Ammonium ion and citric acid supplementation in batch cultures of *Aspergillus niger* B-60. *Biotechnol. Lett.* **14**, 831-836 (1992).

132. Steinbock, F., Choojun, S., Held, I., Roehr, M., & Kubicek, C.P. Characterization and regulatory properties of a single hexokinase from the citric acid accumulating fungus *Aspergillus niger*. *Biochim. Biophys. Acta - Gen. Subj.***1200**, 215-223 (1994).

133. Garg, N. & Hang, Y.D. Microbial production of organic acids from carrot processing waste. *J. Food Sci. Technol.-Mysore* **32**, 119-121 (1995).

134. Legisa, M. & GradisnikGrapulin, M. Sudden substrate dilution induces a higher rate of citric acid production by *Aspergillus niger*. *Appl. Environ. Microbiol.* **61**, 2732-2737 (1995).

135. Legisa, M. & Bencina, M. Evidence for the activation of 6-phosphofructo-1-kinase by cAMP-dependent protein-kinase in *Aspergillus niger*. *FEMS Microbiol. Lett.* **118**, 327-333 (1994).

136. Arisan-Atac, I., Wolschek, M.F., & Kubicek, C.P. Trehalose-6-phosphate synthase A affects citrate accumulation by *Aspergillus niger* under conditions of high glycolytic flux. *FEMS Microbiol. Lett.* **140**, 77-83 (1996).

137. GradisnikGrapulin,M. & Legisa,M. Comparison of specific metabolic characteristics playing a role in citric acid excretion between some strains of the genus *Aspergillus*. *Journal of Biotechnology* **45**, 265-270 (1996).

138. Jernejc, K. & Legisa, M. Purification and properties of carnitine acetyltransferase from citric acid producing *Aspergillus niger*. *Appl. Biochem. Biotechnol.* **60**, 151-158 (1996).

139. Torres, N.V., Riol-Cimas, J.M., Wolschek, M., & Kubicek, C.P. Glucose transport by *Aspergillus niger*: The low affinity carrier is only formed during growth on high glucose concentrations. *Appl. Microbiol. Biotechnol.***44**, 790-794 (1996).

140. Vergano, M.G.F., Soria, M.A., & Kerber, N.L. Influence of inoculum preparation on citric acid production by *Aspergillus niger*. *World J. Microbiol. Biotechnol.* **12**, 655-656 (1996).

141. Gradisnik-Grapulin, M. & Legisa, M. A spontaneous change in the intracellular cyclic AMP level in *Aspergillus niger* is influenced by the sucrose concentration in the medium and by light. *Appl. Environ. Microbiol.* **63**, 2844-2849 (1997).

142. Netik, A., Torres, N.V., Riol, J.M., & Kubicek, C.P. Uptake and export of citric acid by *Aspergillus niger* is reciprocally regulated by manganese ions. *Biochim. Biophys. Acta - Biomemb.* **1326**, 287-294 (1997).

143. Berovic, M. Scale-up of citric acid fermentation by redox potential control. *Biotechnol. Bioeng.* **64**, 552-557 (1999).

144. Legisa, M. & Grdadolnik, S.G. Influence of dissolved oxygen concentration on intracellular pH and consequently on growth rate of *Aspergillus niger*. *Food Technol. Biotechnol.* **40**, 27-32 (2002).

145. Peksel,A., Torres,N.V., Liu,J., Juneau,G., & Kubicek,C.P. C-13-NMR analysis of glucose metabolism during citric acid production by *Aspergillus niger*. *Appl. Microbiol. Biotechnol.* **58**, 157-163 (2002).

146. Peksel, A. & Kubicek, C.P. Effects of sucrose concentration during citric acid accumulation by *Aspergillus niger*. *Turk. J.Chem.* **27**, 581-590 (2003).

147. Berovic, M., Vodopivec, M., & Milicic, S. The influence of manganese ions on *Aspergillus niger* biomass and citric acid biosynthesis in repeated fed batch fermentation. *Chem. Biochem.Eng. Quart.* **20**, 281-284 (2006).

148. Pintado, J., Gonzalez, M.P., & Murado, M.A. Interactions between pretreatment and nutrient concentrations of mussel processing effluents for citric acid production. *Enzyme Microb. Tech.* **20**, 544-549 (1997).

149. Das, A. & Roy, P. Improved production of citric acid by a diploid strain of *Aspergillus niger*. *Can. J. Microbiol.* **24**, 622-625 (1978).

150. Pintado, J., Lonsane, B.K., Gaime-Perraud, I., & Roussos, S. On-line monitoring of citric acid production in solid-state culture by respirometry. *Process Biochem.* **33**, 513-518 (1998).

151. Angumeenal, A.R. & Venkappayya, D. Growth kinetics of heavy metal adapted *Aspergillus niger* during citric acid biosynthesis. *J. Sci. Ind.l Res.* **63**, 610-613 (2004).

152. Gupta, J.K., Heding, L.G., & Jorgensen, O.B. Effect of sugars, hydrogen-ion concentration and ammonium-nitrate on formation of citric acid by *Aspergillus niger*. *Acta Microbiol. Acad. Scient. Hung.* **23**, 63-67 (1976).

153. Vedehra, D.V., Gigia, V., Megh, R., & Gupta, J.K. Inhibitory effect of molasses for citric acid production by *Aspergillus niger*. *Indian J. Exp. Biol.* **15**, 335-336 (1977).

154. Singh, V.K., Vadehra, D.V., & Gupta, J.K. Occurrence of an organic inhibitor in cane molasses affecting citric acid production by *Aspergillus niger*. *Enzyme Microb. Tech.* **3**, 341-343 (1981).

155. Hessing, J.G.M. *et al.* Isolation and characterization of a 1,4-beta-endoxylanase gene of *A. awamori*. *Curr. Genet.* **26**, 228-232 (1994).

156. Gouka, R.J., Hessing, J.G.M., Stam, H., Musters, W., & van den Hondel, C.A.M.J. A Novel Strategy for the isolation of defined *PyrG* mutants and the development of a site-specific integration system for *Aspergillus awamori*. *Curr. Genet.* **27**, 536-540 (1995).

157. Gouka, R.J. *et al.* An expression system based on the promoter region of the *Aspergillus awamori* 1,4-beta-endoxylanase A gene. *Appl. Microbiol. Biotechnol.***46**, 28-35 (1996).

158. Gouka, R.J., Punt, P.J., Hessing, J.G.M. & van den Hondel, C.A.M.J. Analysis of heterologous protein production in defined recombinant *Aspergillus awamori* strains. *Appl. Environ. Microbiol.* **62**, 1951-1957 (1996).

159. Gouka, R.J., van der Heiden, M., Swarthoff, T., & Verrips, C.T. Cloning of a phenol oxidase gene from *Acremonium murorum* and its expression in *Aspergillus awamori*. *Appl. Environ. Microbiol.* **67**, 2610-2616 (2001).

160. Gouka, R.J., Punt, P.J., & van den Hondel, C.A.M.J. Glucoamylase gene fusions alleviate limitations for protein production in *Aspergillus awamori* at the transcriptional and (post)translational levels. *Appl. Environ. Microbiol.* **63**, 488-497 (1997).

161. Joosten, V., Gouka, R.J., van den Hondel, C.A.M.J., Verrips, C.T., & Lokman, B.C. Expression and production of llama variable heavy-chain antibody fragments (V(HH)s) by *Aspergillus awamori*. *Appl. Microbiol. Biotechnol.* **66**, 384-392 (2005).

162. van Gemeren, I.A. *et al.* The effect of pre- and pro-sequences and multicopy integration on heterologous expression of the *Fusarium solani* **pisi** cutinase gene in *Aspergillus awamori*. *Appl. Microbiol. Biotechnol.* **45**, 755-763 (1996).

163. van Gemeren, I.A. *et al.* The ER chaperone encoding bipA gene of black Aspergilli is induced by heat shock and unfolded proteins. *Gene* **198**, 43-52 (1997).

164. Vanhartingsveldt, W., Mattern, I.E., Vanzeijl, .M.J., Pouwels, P.H., & van den Hondel, C.A.M.J. Development of a homologous transformation system for *Aspergillus niger* based on the PyrG gene. *Mol. Gen. Genet.* **206**, 71-75 (1987).

165. Archer, D.B. *et al.* Hen egg-white lysozyme expressed in, and secreted from, *Aspergillus niger* is correctly processed and folded. *Bio-Technology* **8**, 741-745 (1990).

166. Khanh, N.Q., Ruttkowski, E., Leidinger, K., Albrecht, H., & Gottschalk, M. Characterization and expression of a genomic pectin methyl esterase-encoding gene in *Aspergillus niger*. *Gene* **106**, 71-77 (1991).

167. Khanh, N.Q., Leidinger, K., Albrecht, H., Ruttkowski, E., & Gottschalk, M. Effects of promoters on the enhancement of pectin methyl esterase expression in *Aspergillus niger*. *Biotechnol. Lett.* **14**, 1047-1052 (1992).

168. Mattern, I.E. *et al.* Isolation and characterization of m utants of *Aspergillus niger* deficient in extracellular proteases. *Mol. Gen. Genet.***234**, 332-336 (1992).

169. Punt, P.J., Zegers, N.D., Busscher, M., Pouwels, P.H., & van den Hondel, C.A.M.J. Intracellular and Extracellular production of proteins in *Aspergillus* under the control of expression signals of the highly expressed *Aspergillus nidulans gpdA* gene. *J. Biotechnol.* **17**, 19-33 (1991).

170. Harmsen, J.A.M., Kusters van Someren, M.A., & Visser, J. Cloning and expression of a 2nd *Aspergillus niger* pectin lyase gene (*pelA*) - Indications of a pectin lyase gene family in *Aspergillus niger*. *Curr. Genet.* **18**, 161-166 (1990).

171. Kusters van Someren, M. *et al.* Characterization of the *Aspergillus niger pelB* gene - structure and regulation of expression. *Mol. Gen. Genet.***234**, 113-120 (1992).

172. Roberts, I.N. *et al.* Heterologous gene-expression in *Aspergillus niger* - a glucoamylase-porcine pancreatic prophospholipase-A(2) fusion protein is secreted and processed to yield mature enzyme. *Gene* **122**, 155-161 (1992).

173. Broekhuijsen, M.P., Mattern, I.E., Contreras, R., Kinghorn, J.R., & van den Hondel, C.A.M.J. Secretion of heterologous proteins by *Aspergillus niger* - production of active human interleukin-6 in a protease-deficient mutant by Kex2-like processing of a glucoamylase-Hil6 fusion protein. *J. Biotechnol.***31**, 135-145 (1993).

174. Flipphi, M.J.A., Vanheuvel, M., Vanderveen, P., Visser, J., & Degraaff, L.H. Cloning and characterization of the *abfB* gene coding for the major alpha-L-arabinofuranosidase (*Abf B*) of *Aspergillus niger*. *Curr. Genet.* **24**, 525-532 (1993).

175. Flipphi, M.J.A., Panneman, H., Vanderveen, P., Visser, J ., & Degraaff, L.H. Molecular cloning, expression and structure of the endo-1,5-alpha-L-arabinase gene of *Aspergillus niger.* *Appl. Microbiol. Biotechnol.* **40**, 318-326 (1993).

176. Jeenes, D.J., Marczinke, B., Mackenzie, D.A., & Archer, D.B. A truncated glucoamylase gene fusion for heterologous protein secretion from *Aspergillus niger*. *FEMS Microbiol. Lett.* **107**, 267-271 (1993).

177. Flipphi, M.J.A., Visser, J., Vanderveen, P., & Degraaff, L.H. Arabinase gene-expression in *Aspergillus niger* - indications for coordinated regulation. *Microbiology-UK* **140**, 2673-2682 (1994).

178. Verdoes, J.C. *et al.* Characterization of an efficient gene cloning strategy for *Aspergillus niger* based on an autonomously replicating plasmid - cloning of the *nicB* gene of *Aspergillus niger.* *Gene* **146**, 159-165 (1994).

179. Verdoes, J.C. *et al.* Evaluation of molecular and genetic approaches to generate glucoamylase overproducing strains of *Aspergillus niger*. *J. Biotechnol.* **36**, 165-175 (1994).

180. Verdoes, J.C. *et al.* Glucoamylase overexpression in *Aspergillus niger* - molecular genetic analysis of strains containing multiple copies of the *glaA*-gene. *Transgen. Res.* **2**, 84-92 (1993).

181. Verdoes, J.C., Punt, P.J., Stouthamer, A.H., & Van den Hondel, C.A.M.J. The effect of multiple copies of the upstream region on expression of the *Aspergillus niger* glucoamylase-encoding gene. *Gene* **145**, 179-187 (1994).

182. Schrickx, J.M. *et al.* Growth and product formation in chemostat and recycling Cultures by *Aspergillus niger* N402 and a glucoamylase overproducing transformant, provided with multiple copies of the *glaA* gene. *J. Gen. Microbiol.* **139**, 2801-2810 (1993).

183. Mikosch, T., Klemm, P., Gassen, H.G., van den Hondel, C.A.M.J., & Kemme, M. Secretion of active human mucus proteinase inhibitor by *Aspergillus niger* after KEX2-like processing of a glucoamylase-inhibitor fusion protein. *J. Biotechnol.* **52**, 97-106 (1996).

184. Punt, P.J. *et al.* Analysis of the role of the gene bipA, encoding the major endoplasmic reticulum chaperone protein in the secretion of homologous and heterologous proteins in black Aspergilli. *Appl. Microbiol. Biotechnol.* **50**, 447-454 (1998).

185. Swift, R.J., Wiebe, M.G., Robson, G.D., & Trinci, A.P.J. Recombinant glucoamylase production by *Aspergillus niger* B1 in chemostat and pH auxostat cultures. *Fungal Genet. Biol.* **25**, 100-109 (1998).

186. Mainwaring, D.O. *et al.* Effect of pH on hen egg white lysozyme production and evolution of a recombinant strain of *Aspergillus niger*. *J. Biotechnol.***75**, 1-10 (1999).

187. Spencer, A. *et al.* Expression, purification, and characterization of the recombinant calcium-binding equine lysozyme secreted by the filamentous fungus *Aspergillus niger*: Comparisons with the production of hen and human lysozymes. *Prot. Expr. Purif.* **16**, 171-180 (1999).

188. Conesa, A., Weelink, G., van den Hondel, C.A.M.J., & Punt, P.J. C-terminal propeptide of the *Caldariomyces fumago* chloroperoxidase: an intramolecular chaperone? *FEBS Lett.* **503**, 117-120 (2001).

189. Conesa, A., Jeenes, D., Archer, D.B., van den Hondel, C.A.M.J., & Punt, P.J. Calnexin overexpression increases manganese peroxidase production in *Aspergillus niger*. *Appl. Environ. Microbiol.* **68**, 846-851 (2002).

190. Conesa, A. *et al.* Expression of the *Caldariomyces fumago* chloroperoxidase in *Aspergillus niger* and characterization of the recombinant enzyme. *J. Biol. Chem.* **276**, 17635-17640 (2001).

191. Conesa, A., van den Hondel,C.A.M.J., & Punt,P.J. Studies on the production of fungal peroxidases in *Aspergillus niger*. *Appl. Environ. Microbiol.* **66**, 3016-3023 (2000).

192. Gordon, C.L. *et al.* Glucoamylase :: green fluorescent protein fusions to monitor protein secretion in *Aspergillus niger*. *Microbiology-Sgm* **146**, 415-426 (2000).

193. Gordon, C.L. *et al.* A glucoamylase :: GFP gene fusion to study protein secretion by individual hyphae of *Aspergillus niger*. *J. Microbiol. Meth.* **42**, 39-48 (2000).

194. Krasevec, N., van den Hondel, C.A.M.J., & Komel, R. Can hTNF-alpha be successfully produced and secreted in filamentous fungus *Aspergillus niger*? *Pflugers Arc. -Eur. J. Physiol.***439**, R84-R86 (2000).

195. Krasevec, N., Svetina, M., Gaberc-Porekar, V., Menart, V., & Komel, R. In vivo and in vitro cleavage of glucoamylase-TNF alpha fusion protein secreted from *Aspergillus niger*. *Food Technol. Biotechnol.***41**, 345-351 (2003).

196. Krasevec, N. & Komel, R. Influence of primary gene structure on secretion of recombinant TNF alpha from *Aspergillus niger.* *Acta Chim. Sloven.* **55**, 350-S359 (2008).

197. Svetina,M., Krasevec,N., Gaberc-Porekar,V., & Komel,R. Expression of catalytic subunit of bovine enterokinase in the filamentous fungus *Aspergillus niger*. *J. Biotechnol.* **76**, 245-251 (2000).

198. Morozova-Roche, L.A. *et al.* Amyloid fibril formation and seeding by wild-type human lysozyme and its disease-related mutational variants. *J.Struct.Biol.***130**, 339-351 (2000).

199. Ngiam, C., Jeenes, D.J., Punt, P.J., van den Hondel, C.A.M.J., & Archer, D.B. Characterization of a foldase, protein disulfide isomerase A, in the protein secretory pathway of *Aspergillus niger*. *Appl. Environ. Microbiol.* **66**, 775-782 (2000).

200. Swift,R.J. *et al.* The effect of organic nitrogen sources on recombinant glucoamylase production by *Aspergillus niger* in chemostat culture. *Fungal Genet. Biol.* **31**, 125-133 (2000).

201. Watson, A.J., Worley, J., Elliott, R.M., Jeenes, D.J., & Archer, D.B. Cloning stress-induced genes from *Aspergillus niger* using polymerase chain reaction-augmented subtractive hybridization. *Anal.l Biochem.* **277**, 162-165 (2000).

202. Wallis,G.L.F. *et al.* The effect of pH on glucoamylase production, glycosylation and chemostat evolution of *Aspergillus niger*. *Biochim.Biophys.Acta - Gen.Subj.* **1527**, 112-122 (2001).

203. Canet, D. *et al.* Local cooperativity in the unfolding of an amyloidogenic variant of human lysozyme. *Nature Structural Biology* **9**, 308-315 (2002).

204. Record, E. *et al.* Expression of the *Pycnoporus cinnabarinus* laccase gene in *Aspergillus niger* and characterization of the recombinant enzyme. *Eur. J. Biochem.* **269**, 602-609 (2002).

205. Valkonen, M., Ward, M., Wang, H.M., Penttila, M., & Saloheimo, M. Improvement of foreign-protein production in *Aspergillus niger* var. *awamori* by constitutive induction of the unfolded-protein response. *Appl. Environ. Microbiol.* **69**, 6979-6986 (2003).

206. Wang, H. *et al.* Isolation and characterisation of a calnexin homologue, clxA, from Aspergillus niger. *Mol. Gen. Genom.* **268**, 684-691 (2003).

207. Ward, M. *et al.* Characterization of humanized antibodies secreted by *Aspergillus niger*. *Appl. Environ. Microbiol.* **70**, 2567-2576 (2004).

208. Talabardon, M. & Yang, S.T. Production of GFP and glucoamylase by recombinant *Aspergillus niger:* Effects of fermentation conditions on fungal morphology and protein secretion. *Biotechnol.Prog.* **21**, 1389-1400 (2005).

209. Guillemette, T. *et al.* Genomic analysis of the secretion stress response in the enzyme-producing cell factory *Aspergillus niger*. *BMC Genom.* **8**, (2007).

210. Punt, P.J. *et al.* Characterization of the *Aspergillus niger* *prtT*, a unique regulator of extracellular protease encoding genes. *Fungal Genet. Biol.* **45**, 1591-1599 (2008).

211. Yuan, X.L. *et al.* *Aspergillus niger* genome-wide analysis reveals a large number of novel alpha-glucan acting enzymes with unexpected expression profiles. *Mol. Gen. Genom.* **279**, 545-561 (2008).

212. Hidayat, B.J., Eriksen, N.T., & Wiebe, M.G. Acid phosphatase production by *Aspergillus niger* N402A in continuous flow culture. *FEMS Microbiol. Lett.* **254**, 324-331 (2006).

213. Dave, A., Jeenes, D.J., Mackenzie, D.A., & Archer, D.B. HaCAV-Independent induction of chaperone-encoding gene bipA in *Aspergillus niger* strains overproducing membrane proteins. *Appl. Environ. Microbiol.* **72**, 953-955 (2006).

214. Fowler,T ., Berka, R.M., & Ward, M. Regulation of the *glaA* gene of *Aspergillus niger*. *Curr. Genet.* **18**, 537-545 (1990).

215. Ward, M., Kodama, K.H., & Wilson ,L.J. Transformation of *Aspergillus awamori* and *A. niger* by Electroporation. *Exp. Mycol.* **13**, 289-293 (1989).

216. Berka, R.M. *et al.* Molecular cloning and deletion of the gene encoding aspergillopepsin A from *Aspergillus awamori*. *Gene* **86**, 153-162 (1990).

217. Korman,D.R. *et al.* Cloning, Characterization, and expression of 2 alpha-amylase genes from *Aspergillus niger* var. *awamori*. *Curr. Genet.* **17**, 203-212 (1990).

218. Dunn-Coleman, N.S. *et al.* Commercial levels of chymosin production by *Aspergillus*. *Bio-Technology* **9**, 976-981 (1991).

219. Ward, M., Wilson, L.J., Kodama, K.H., Rey, M.W., & Berka, R.M. Improved Production of chymosin in *Aspergillus* by expression as a glucoamylase-chymosin fusion. *Bio-Technology* **8**, 435-440 (1990).

220. Ward, M., Wilson, L.J., & Kodama, K.H. Use of *Aspergillus* overproducing mutants, cured for integrated plasmid, to overproduce heterologous proteins. *Appl. Microbiol. Biotechnol.* **39**, 738-743 (1993).

221. Ward, P.P. *et al.* A system for production of commercial quantities of human lactoferrin - a broad-spectrum natural antibiotic. *Bio-Technology* **13**, 498-503 (1995).

222. Lombrana, M., Moralejo, F.J., Pinto, R., & Martin, J.F. Modulation of *Aspergillus awamori* thaumatin secretion by modification of *bipA* gene expression. *Appl. Environ. Microbiol.* **70**, 5145-5152 (2004).

223. Kappeler, S.R. *et al.* Characterization of recombinant camel chymosin reveals superior properties for the coagulation of bovine and camel milk. *Biochem. Biophys. Res. Com.* **342**, 647-654 (2006).

224. de Vries, R.P. *et al.* A New Black *Aspergillus s*pecies, *A. vadensis*, is a promising host for homologous and heterologous protein production. *Appl. Environ. Microbiol.* **70**, 3954-3959 (2004).

225. Silman, R.W., Black, L.T., Mcghee, J.E., & Bagley, E.B. Hydrolysis of raffinose in a hollow-fiber reactor using an unrefined mixture of alpha-galactosidase and invertase. *Biotechnol. Bioeng.* **22**, 533-541 (1980).

226. Allen, A. & Sternberg, D. Beta-glucosidase production by *Aspergillus phoenicis* in stirred-tank fermentors. *Biotechnol. Bioeng.* **22**, 189-197 (1980).

227. Bussink, H.J.D., Vandenhombergh, J.P.T.W., Vandenijssel, P.R.L.A., & Visser, J. Characterization of polygalacturonase-overproducing *Aspergillus niger* transformants. *Appl. Microbiol. Biotechnol.* **37**, 324-329 (1992).

228. Bussink, H.J.D., Brouwer, K.B., Degraaff, L.H., Kester, H.C.M., & Visser, J. Identification and characterization of a 2nd polygalacturonase gene of *Aspergillus niger*. *Curr. Genet.* **20**, 301-307 (1991).

229. Bussink, H.J.D., Kester, H.C.M., & Visser, J. Molecular cloning, nucleotide-sequence and expression of the gene encoding prepro-polygalacturonase ii of *Aspergillus niger*. *FEBS Lett.* **273**, 127-130 (1990).

230. Bussink, H.J.D., Buxton, F.P., Fraaye, B.A., Degraaff, L.H., & Visser, J. The polygalacturonases of *Aspergillus niger* are encoded by a family of diverged genes. *Eur. J.Biochem.* **208**, 83-90 (1992).

231. Jarai,G., Kirchherr,D., & Buxton,F.P. Cloning and Characterization of the Pepd Gene of *Aspergillus niger* which codes for A subtilisin-Like Protease. *Gene* **139**, 51-57 (1994).

232. Jarai, G. & Buxton, F. Nitrogen, carbon, and pH regulation of extracellular acidic proteases of *Aspergillus niger*. *Curr. Genet.* **26**, 238-244 (1994).

233. Faulds, C.B., deVries, R.P., Kroon, P.A., Visser, J., & Williamson, G. Influence of ferulic acid on the production of feruloyl esterases by *Aspergillus niger*. *FEMS Microbiol. Lett.* **157**, 239-244 (1997).

234. Faulds, C.B. & Williamson, G. Purification and Characterization of a ferulic acid esterase (Fae-Iii) from *Aspergillus niger* - specificity for the phenolic moiety and binding to microcrystalline cellulose. *Microbiology-UK* **140**, 779-787 (1994).

235. Faulds, C.B., Kroon, P.A., Saulnier, L., Thibault, J.F., & Williamson, G. Release of ferulic acid from maize bran and derived oligosaccharides by *Aspergillus niger* esterases. *Carbohydrate Polymers* **27**, 187-190 (1995).

236. Faulds, C.B. & Williamson, G. Release of ferulic acid from wheat bran by a ferulic acid esterase (Fae-Iii) from *Aspergillus niger*. *Appl. Microbiol. Biotechnol.* **43**, 1082-1087 (1995).

237. Frederick, K.R. *et al.* Glucose-oxidase from *Aspergillus niger* - cloning, gene sequence, secretion from *Saccharomyces cerevisiae* and kinetic analysis of a yeast-derived enzyme. *J. Biol. Chem.* **265**, 3793-3802 (1990).

238. de Groot, M.J.L. *et al.* Regulation of pentose catabolic pathway genes of *Aspergillus niger*. *Food Technol. Biotechnol.***45**, 134-138 (2007).

239. de Vries, J.D., Rao, K.S., & Willis, R.H. Application of a radioreceptor assay to the screening and characterisation of compounds from marine organisms with activity at the phorbol ester binding site of protein kinase C. *Toxicon* **35**, 347-354 (1997).

240. de Vries, R.P. *et al.* Expression profiling of pectinolytic genes from *Aspergillus niger*. *FEBS Lett.* **530**, 41-47 (2002).

241. de Vries, R.P. & Visser, J . Regulation of the feruloyl esterase (*faeA*) gene from *Aspergillus niger*. *Appl. Environ. Microbiol.* **65**, 5500-5503 (1999).

242. de Vries, R.P., Vankuyk, P.A., Kester, H.C.M., & Visser, J. The *Aspergillus niger faeB* gene encodes a second feruloyl esterase involved in pectin and xylan degradation and is specifically induced in the presence of aromatic compounds. *Biochem. J.* **363**, 377-386 (2002).

243. de Vries, R.P. *et al.* The beta-1,4-endogalactanase A gene from *Aspergillus niger* is specifically induced on arabinose and galacturonic acid and plays an important role in the degradation of pectic hairy regions. *Eur. J. Biochemistry* **269**, 4985-4993 (2002).

244. van Peij, N.N.M.E., Gielkens, M.M.C., de Vries, R.P., Visser, ., & de Graaff, L.H. The transcriptional activator *xlnR* regulates both xylanolytic and endoglucanase gene expression in *Aspergillus niger*. *Appl. Environ. Microbiol.* **64**, 3615-3619 (1998).

245. Versaw, W.K., Bevins, M.A., & Markwell,J . Purification and properties of a 4-nitrophenylphosphatase from *Aspergillus niger*. *Arch. Biochem.Biophys.* **287**, 85-90 (1991).

246. Vanderveen, P., Flipphi, M.J.A., Voragen, A.G.J., & Visser, J. Induction, purification and characterization of arabinases produced by *Aspergillus niger*. *Arch. Microbiol.* **157**, 23-28 (1991).

247. Gottschalk, T.E., Nielsen, J.E., & Rasmussen, P. Detection of endogenous beta-glucuronidase activity in *Aspergillus niger*. *Appl.Microbiol.Biotechnol.***45**, 240-244 (1996).

248. Martens-Uzunova, E.S. & Schaap, P.J. Assessment of the pectin degrading enzyme network of *Aspergillus niger* by functional genomics. *Fungal Genet. Biol.* **46**, S170-S179 (2009).

249. Adham, N.Z. & Ahmed, E.M. Extracellular lipase of *Aspergillus niger* NRRL3; production, partial purification and properties. *Ind. J.Microbiol.* **49**, 77-83 (2009).

250. Smith, P.T., King, A.D., & Goodman, N. Isolation and characterization of urease from *Aspergillus niger*. *J. Gen. Microbiol.* **139**, 957-962 (1993).

251. Prasertsan, P., Kittikun, A.H., Kunghae, A., Maneesri, J., & Susumu, O. Optimization for xylanase and cellulase production from *Aspergillus niger* ATCC 6275 in palm oil mill wastes and its application. *World J Microbiol Biotechnol* **13**, 555-559 (1997).

252. Ong, L.G.A., Abd-Aziz, S., Noraini, S., Karim, M.I.A., & Hassan, M.A. Enzyme production and profile by *Aspergillus niger* - During solid substrate fermentation using palm kernel cake as substrate. *Appl. Biochem. Biotechnol.* **118**, 73-79 (2004).

253. D'Annibale, A., Sermanni, G.G., Federici, F., & Petruccioli, M. Olive-mill wastewaters: a promising substrate for microbial lipase production. *Biores. Technol.* **97**, 1828-1833 (2006).

254. Lemense, E.H., Corman,J ., Vanlanen, J.M., & Langlykke, A.F. Production of mold amylases in submerged culture. *J. Bacteriol.* **54**, 149-159 (1947).

255. Bloch,F., Brown,G.E., & Farkas,D.F. Utilization of alkaline potato peel waste by fermentation - amylase production by *Aspergillus foetidus* NRRL337, and alcoholic fermentation. *Amer. Potato J.* **50**, 357-364 (1973).

256. Iwai, M., Okumura, S., Deleal, E.L., & Tsujisaka,Y. Studies on microbial esterases .1. Purification of 4 esterases from *Aspergillus niger* NRRL-337. *Agric. Biol. Chem.* **47**, 1865-1868 (1983).

257. Aguero, J.M.Z., Demacedo, G.R., Facciotti, M.C.R., & Schmidell, W. Influence of pH on glucoamylase synthesis and secretion by *Aspergillus awamori* NRRL 3112 and *Aspergillus niger* NRRL-337. *Rev. Microbiol.* **21**, 355-360 (1990).

258. Hang, Y.D. & Woodams, E.E. Apple pomace - a potential substrate for production of beta-glucosidase by *Aspergillus foetidus*. *Lebensm. Wiss. Technol.* **27**, 587-589 (1994).

259. Hours, R.A., Voget, C.E., & Ertola, R.J. Some factors affecting pectinase production from apple pomace in solid-state cultures. *Biol. Wastes* **24**, 147-157 (1988).

260. Garzon, C.G. & Hours, R.A. Citrus waste - an alternative substrate for pectinase production in solid-state culture. *Biores. Technol.***39**, 93-95 (1992).

261. Cavalitto, S.F., Arcas, J.A., & Hours, R.A. Pectinase production profile of *Aspergillus foetidus* in solid state cultures at different acidities. *Biotechnol. Lett.***18**, 251-256 (1996).

262. Sogi, D.S., Gupta, U., Garg, S.K., & Bawa, A.S. Utilization of tomato seeds isolated from processing waste for enzyme production using *Aspergillus niger*. *J. Food Science . Technol.-Mysore* **41**, 207-210 (2004).

263. Vialta, A. & Bonatelli, R. Improved glucoamylase production by less unstable strains of *Aspergillus awamori*. *Biotechnol. Lett.* **10**, 737-740 (1988).

264. Han, I.Y. & Steinberg, M.P. Amylolysis of raw corn by *Aspergillus niger* for simultaneous ethanol fermentation. *Biotechnol. Bioeng.* **30**, 225-232 (1987).

265. Dartora, A.B., Bertolin, T.E., Bilibio, D., Silveira, M.M., & Costa, J.A.V. Evaluation of filamentous fungi and inducers for the production of endo-polygalacturonase by solid state fermentation. *Z. Naturforsch. C- J. Biosci.* **57**, 666-670 (2002).

266. Costa, J.A.V. Estudo da produção de amiloglucosidase por *Aspergillus niger* NRRL 3122 em fermentação semi-sólida de farelo de arroz. -1. 1996. 203, Thesis (Food Eng. Degree), Unicamp, São Paulo, Brazil.

267. Costa, J.A.V., Alegre, R.M., & Hasan, S.D.M. Packing density and thermal conductivity determination for rice bran solid-state fermentation. *Biotechnol. Techniq.* **12**, 747-750 (1998).

268. Silveira, S.T., Oliveira, M.S., Costa, J.A.V., & Kalil, S.J. Optimization of glucoamylase production by *Aspergillus niger* in solid-state fermentation. *Appl .Biochem. Biotechnol.* **128**, 131-139 (2006).

269. Desgranges, C. & Durand, A. Effect of Pco2 on growth, conidiation, and enzyme-production in solid-state culture on *Aspergillus niger* and *Trichoderma viride* TS. *Enzyme Microb. Tech.* **12**, 546-561 (1990).

270. Manera, A.P., Kamimura, E.S., Brites, L.M., & Kalil, S.J. Adsorption of amyloglucosidase from *Aspergillus niger* NRRL 3122 using ion exchange resin. *Braz. Arch. Biol. Technol.* **51**, 1015-1024 (2008).

271. Rizzatto, L. Estudo de produção de pectinases per *Penicillium italicum* IZ 1584 e *Aspergillus niger* NRRL 3122 por fermentação semi-sólida em bagaço de laranja industrializado. -89. 1999. Master thesis. Faculdade de Engenharia de Alimentos. Universidade de Estadual de Campinas, Brazil.

272. Van Dijck, P.W., Selten, G.C., & Hempenius, R.A. On the safety of a new generation of DSM *Aspergillus niger* enzyme production strains. *Regul. Toxicol. Pharmacol.* **38**, 27-35 (2003).

273. Ullah, A.H.J. & Gibson, D.M. Extracellular Phytase (Ec 3.1.3.8) from *Aspergillus ficuum* NRRL 3135 - Purification and Characterization. *Prep. Biochem.* **17**, 63-91 (1987).

274. Ebune, A., AlAsheh, S., & Duvnjak, Z. Effects of phosphate, surfactants and glucose on phytase production and hydrolysis of phytic acid in canola meal by *Aspergillus ficuum* during solid-state fermentation. *Biores. Technol.* **54**, 241-247 (1995).

275. Ebune, A., AlAsheh, S., & Duvnjak, Z. Production of phytase during solid-state fermentation using *Aspergillus ficuum* NRRL 3135 in canola-meal. *Biores. Technol.* **53**, 7-12 (1995).

276. Mccrae, S.I., Leith, K.M., Gordon, A.H., & Wood, T.M. Xylan-degrading enzyme-system produced by the fungus *Aspergillus awamori* - Isolation and characterization of a feruloyl esterase and a p-coumaroyl esterase. *Enzyme Microb. Tech.* **16**, 826-834 (1994).

277. Pertino, M., Schmeda-Hirschmann, G., Santos, L.S., Rodriguez, J.A., & Theoduloz, C. Biotransformation of jatrophone by *Aspergillus niger* ATCC 16404. *Z. Naturforsch. Section B - J. Chem. Sci.* **62**, 275-279 (2007).

278. Schmeda-Hirschmann, G., Astudillo, L., & Palenzuela, J.A. Biotransformation of solidagenone by *Alternaria alternata, Aspergillus niger* and *Curvularia lunata* cultures. *World J. Microbiol. Biotechnol.* **20**, 93-97 (2004).

279. Schmeda-Hirschmann, G., Aranda, C., Kurina, M., Rodriguez, J.A., & Theoduloz, C. Biotransformations of imbricatolic acid by *Aspergillus niger* and *Rhizopus nigricans* cultures. *Molecules* **12**, 1092-1100 (2007).

280. Seo, J. *et al.* Biodegradation of the insecticide N,N-diethyl-m-toluamide by fungi: Identification and toxicity of metabolites. *Arch. Env. Contam. Tox.* **48**, 323-328 (2005).

281. Toniazzo, G., De Oliveira, D., Dariva ,C., Oestreicher, E.G., & Antunes, O.A.C. Biotransformation of (-)beta-pinene by *Aspergillus niger* ATCC 9642. *Appl. Biochem. Biotechnol.* **121**, 837-844 (2005).

282. Kiran, I., Yildirim, H.N., Hanson, J.R., & Hitchcock, P.B. The antifungal activity and biotransformation of diisophorone by the fungus *Aspergillus niger*. *J. Chem. Tech. Biotechnol.* **79**, 1366-1370 (2004).

283. Gouiric, S.C. *et al.* 1 beta,7 beta-Dihydroxydehydroabietic acid, a new biotransformation product of dehydroabietic acid by *Aspergillus niger.* *World J. Microbiol. Biotechnol.* **20**, 281-284 (2004).

284. Chou, B.H. *et al.* Microbial transformation of isosteviol lactone and evaluation of the transformation products on androgen response element. *J. Nat. Prod.* **71**, 602-607 (2008).

285. Yang, L.M. *et al.* Microbial metabolism of steviol and steviol-16 alpha,17-epoxide. *Phytochemistry* **68**, 562-570 (2007).

286. Myung, K., Manthey, J.A., & Narciso,J .A. *Aspergillus niger* metabolism of citrus furanocoumarin inhibitors of human cytochrome P450 3A4. *Appl. Microbiol. Biotechnol.* **78**, 343-349 (2008).

287. Bisogno, F.R. *et al.* Atypical regioselective biohydrolysis on steroidal oxiranes by *Aspergillus niger* whole cells: Some stereochemical features. *Steroids* **72**, 643-652 (2007).

288. Metwally, M. *et al.* Bioenergetic consequences of glucoamylase production in carbon-limited chemostat cultures of *Aspergillus niger*. *Anton Leeuw Int J G* **59**, 35-43 (1991).

289. Poje, M., Nota, O., & Balenovic, K. Stereoselective oxidation of gem-disulfides with *Aspergillus niger*. *Tetrahedron* **36**, 1895-1897 (1980).

290. Adham, N.Z., Zaki, R.A., & Naim, N. Microbial transformation of diosgenin and its precursor furostanol glycosides. *World J. Microbiol. Biotechnol.* **25**, 481-487 (2009).

291. Demyttenaere, J.C.R., Adams, A., Vanoverschelde, J., & De Kimpe, N. Biotransformation of (S)-(+)-linalool by *Aspergillus niger*: An investigation of the culture conditions. *J. Agric. Food Chem.* **49**, 5895-5901 (2001).

292. Demyttenaere, J.C.R. & Willemen, H.M. Biotransformation of linalool to furanoid and pyranoid linalool oxides by *Aspergillus niger*. *Phytochemistry* **47**, 1029-1036 (1998).

293. Aranda, G. *et al.* A new example of 1 alpha-hydroxylation of drimanic terpenes through combined microbial and chemical processes. *Tetrahedron* **57**, 6051-6056 (2001).

294. Chen, A.R.M. & Reese, P.B. Biotransformation of terpenes from Stemodia maritima by *Aspergillus niger* ATCC 9142. *Phytochem.* **59**, 57-62 (2002).

295. He, A. & Rosazza, J.P.N. Microbial transformations of S-naproxen by *Aspergillus niger* ATCC 9142. *Pharmazie* **58**, 420-422 (2003).

296. Mirata, M.A., Wust, M., Mosandl, A., & Schrader, J. Fungal biotransformation of (+/-)-linalool. *J. Agric. Food Chem.* **56**, 3287-3296 (2008).

297. Grivel, F., Larroche, C., & Gros, J.B. Determination of the reaction yield during biotransformation of the volatile and chemically unstable compound beta-ionone by *Aspergillus niger.* *Biotechnol. Prog.* **15**, 697-705 (1999).

298. Ata, A., Conci, L.J., Betteridge, J., Orhan, I., & Sener, B. Novel microbial transformations of sclareolide. *Chem. Pharm. Bull.* **55**, 118-123 (2007).

299. Papanikolau S, Dimou A, Fakas S, Diamantopoulos P, Phillipousis A, Galiotou-Penayotou M, & Angelis G. Biotechnological conversion of waste cooking oil into lipid-rich biomass. *J. Appl. Microbiol.* **110**: 1138-1150 (2011).
