# Supplementary material for: Fumonisin and Ochratoxin Production in Industrial Aspergillus niger Strains
Source: PLoS One. 2011 Aug 11;6(8):e23496. doi: 10.1371/journal.pone.0023496 (PMC3154942; doi:10.1371/journal.pone.0023496)
Supplement: Table S5 — contains the results of citric acid production, fumonsin, ochratoxin A nad qualitative malformin production on the medium CIT4 by 9 strains of A. niger. (DOC) [file pone.0023496.s005.doc]

**Table S5**. Supplementary material.

Fumonisin and ochratoxin production in industrial *Aspergillus niger* strains.

by

Jens C. Frisvad, Thomas O. Larsen, Ulf Thrane, Martin Meijer, Janos Varga, Robert A. Samson & Kristian Fog Nielsen

**Table S5**. Citric acid, ochratoxin A, fumonisin, and malformin production and degree of sporulation on CIT4 medium under still and shake conditions by 9 strains of *Aspergillus niger*. Cultures were grown at 30°C for shake cultures and at 25°C for still cultures and incubated for 8 days.

| Strain | Citric acid (g/l) | Ochra-toxin A (ng/ml) broth / mycelium | Fumo-nisin B_2_ (ng/ml) | Fumo-nisin B_2_ in myce-lium (ng/g) | Fumo-nisin B_4_ | Mal-formin C1 and C2 | Mal-formin A1 | Sporu-  lation  Scale:  0-3 |
| --- | --- | --- | --- | --- | --- | --- | --- | --- |
| CBS^a^ 101705 | 24.6^b^ | 0 / 28^c^ | 63^c^ | 131^c^ | +^d^ | +^d^ | +^d^ | 0 |
| CBS 101705  shake | 24.2 | 0 / 68 | 2 | 308 | + | + | - | 1.5 |
| CBS 101705 | 16.7 | 14 / 1101 | 66 | 1021 | + | + | + | 0.5 |
| CBS 101705  shake | 22.8 | 90 / 1352 | 6 | 526 | + | + | + | 1 |
| IBT 19558 | 12.7 | 0 / 0 | 0 | 0 | - | + | + | 2 |
| IBT 19558  shake | 11.8 | 0 / 0 | 0 | 3.5 | - | + | - | 2 |
| NRRL 3122 | 23.6 | 0 / 4 | 0 | 0 | - | + | + | 1 |
| NRRL 3122  shake | 19.8 | 0 / 0 | 0 | 0 | - | + | + | 0.5 |
| NRRL 3 | 22.3 | 0 / 0 | 27.3 | 506 | + | trace | - | 2 |
| NRRL 3  shake | 23.8 | 0 / 0 | 39.5 | 876 | + | trace | - | 2 |
| CBS 126.48 | 1.8 | 115 / 2228 | 0 | 1.5 | - | + | + | 0 |
| CBS 126.48  shake | 1.7 | 71 / 2085 | 0 | 0 | - | + | + | 0 |
| NRRL 567 | 19.5 | 0 / 0 | 6382 | 225 | + | + | + | 1 |
| NRRL 567 shake | 16.0 | 0 / 0 | 160 | 3352 | + | + | + | 1.5 |
| NRRL 599 | 27.3 | 0 / 0 | 12.5 | 382 | + | - | - | 0 |
| NRRL 599  shake | 33.9 | 0 /0 | 0 | 550 | + | - | - | 0.5 |
| NRRL 330 | 27.1 | 0 / 0 | 3.5 | 337 | - | + | + | 1 |
| NRRL 330 shake | 19.8 | 0 / 0 | 4.2 | 298 | - | + | + | 2 |
| NRRL 350 | 25.1 | 0 / 0 | 34.5 | 868 | + | trace | - | 1 |
| NRRL 350  shake | 24.8 | 0 / 0 | 0 | 673 | + | trace | - | 3 |

^a^ Underlined strains can produce OTA in YES agar and several other agar media.

^b^ Citric acid determinations were not duplicated, but values in still and shake cultures with the same fungus & medium were comparable.

^c^ Values are averages of two biological replicates. Despite the quantification the main purpose here was to show that the mycotoxins were produced and the approximate level of mycotoxin, with less emphasis on reproducibility.

^d^ The values were not quantified because of lack of sufficient amounts of pure authentic standards
